# STIM1 is a metabolic checkpoint regulating the invasion and metastasis of hepatocellular carcinoma

**DOI:** 10.7150/thno.44025

**Published:** 2020-05-16

**Authors:** Huakan Zhao, Guifang Yan, Lu Zheng, Yu Zhou, Halei Sheng, Lei Wu, Qi Zhang, Juan Lei, Jiangang Zhang, Rong Xin, Lu Jiang, Xiao Zhang, Yu Chen, Jingchun Wang, Yanquan Xu, Dingshan Li, Yongsheng Li

**Affiliations:** 1 Clinical Medicine Research Center, Xinqiao Hospital, Army Medical University, Chongqing 400037, China.; 2 Institute of Cancer, Xinqiao Hospital, Army Medical University, Chongqing 400037, China.; 3 Department of Hepatobiliary Surgery, Xinqiao Hospital, Army Medical University, Chongqing 400037, China.

**Keywords:** invasion and metastasis, metabolic reprogramming, Snail1, SOCE, STIM1

## Abstract

**Background**: Cancer cells undergoing invasion and metastasis possess a phenotype with attenuated glycolysis, but enhanced fatty acid oxidation (FAO). Calcium (Ca^2+^)-mediated signaling pathways are implicated in tumor metastasis and metabolism regulation. Stromal-interaction molecule 1 (STIM1) triggered store-operated Ca^2+^ entry (SOCE) is the major route of Ca^2+^ influx for non-excitable cells including hepatocellular carcinoma (HCC) cells. However, whether and how STIM1 regulates the invasion and metastasis of HCC *via* metabolic reprogramming is unclear.

**Methods**: The expressions of STIM1 and Snail1 in the HCC tissues and cells were measured by immunohistochemistry, Western-blotting and quantitative PCR. STIM1 knockout-HCC cells were generated by CRISPR-Cas9, and gene-overexpression was mediated *via* lentivirus transfection. Besides, the invasive and metastatic activities of HCC cells were assessed by transwell assay, anoikis rate* in vitro* and lung metastasis *in vivo*. Seahorse energy analysis and micro-array were used to evaluate the glucose and lipid metabolism.

**Results**: STIM1 was down-regulated in metastatic HCC cells rather than in proliferating HCC cells, and low STIM1 levels were associated with poor outcome of HCC patients. During tumor growth, STIM1 stabilized Snail1 protein by activating the CaMKII/AKT/GSK-3β pathway. Subsequently, the upregulated Snail1 suppressed STIM1/SOCE during metastasis. STIM1 restoration significantly diminished anoikis-resistance and metastasis induced by Snail1. Mechanistically, the downregulated STIM1 shifted the anabolic/catabolic balance, *i.e.*, from aerobic glycolysis towards AMPK-activated fatty acid oxidation (FAO), which contributed to Snail1-driven metastasis and anoikis-resistance.

**Conclusions**: Our data provide the molecular basis that STIM1 orchestrates invasion and metastasis *via* reprogramming HCC metabolism.

## Introduction

Hepatocellular carcinoma (HCC) is one of the most common malignancies and the third leading cause of cancer-related mortalities owing to its high metastatic rate 1. The epithelial-mesenchymal transition (EMT) is pivotal for the invasion and metastasis of cancer cells 2. Cancer cells acquire energy and material basis for rapid tumor growth by enhanced anabolism, while the EMT tumor cells depend on catabolic pathways to survive from metabolic stress during metastasis 3-5. However, the metabolic reprogramming during the invasion and metastasis of HCC cells is still unknown. Exploring the underlying mechanism is critical for developing efficient strategies for preventing HCC metastasis.

Calcium (Ca^2+^)-mediated signaling pathways are implicated in tumorigenesis and metastasis, and Ca^2+^ is finely regulated within cellular compartments to sense signaling pathways to precisely respond to various stimuli 6. Stromal interaction molecule 1 (STIM1), as an endoplasmic reticulum (ER) Ca^2+^ sensor, triggers store-operated Ca^2+^ entry (SOCE), which is the major route of Ca^2+^ influx for non-excitable cells including HCC cells 7, 8. We previously reported that STIM1 is upregulated during tumor growth and correlates with elevated hypoxia-inducible factor-1 alpha (HIF-1α) in hypoxic HCC. HIF-1 promotes STIM1 mRNA synthesis and induces SOCE, which in return stabilizes HIF-1α by activating Ca^2+^/calmodulin-dependent protein kinase II (CaMKII) 9. Recently, emerging evidence indicates that STIM1-mediated SOCE is closely related to metabolic regulation. For example, STIM1 regulates the cell-cycle and proliferation of activated T cells by upregulating glycolysis and oxidative phosphorylation (OXPHOS) 10. SOCE promotes lipolysis *via* cyclic adenosine monophosphate (cAMP)-dependent upregulation of peroxisome proliferator activated receptor (PPAR) alpha in skeletal myofibers 11. Cardiomyocytes lacking STIM1 exhibits dysregulated cardiac glucose and lipid metabolism 12. Although STIM1-mediated SOCE is essential for the migration of various cell types, including tumor cells 13-15, the role of STIM1 in dynamic HCC progression, especially in metastatic HCC cells, remains unclear.

In this study, we aimed to explore the role of STIM1 in the metabolic reprogramming of metastatic and proliferative HCC cells. Our results may highlight a potential therapeutic target for the pathogenesis and metastatic progression of HCC.

## Results

### STIM1 is downregulated in metastatic HCC cells

**Figure 1:**
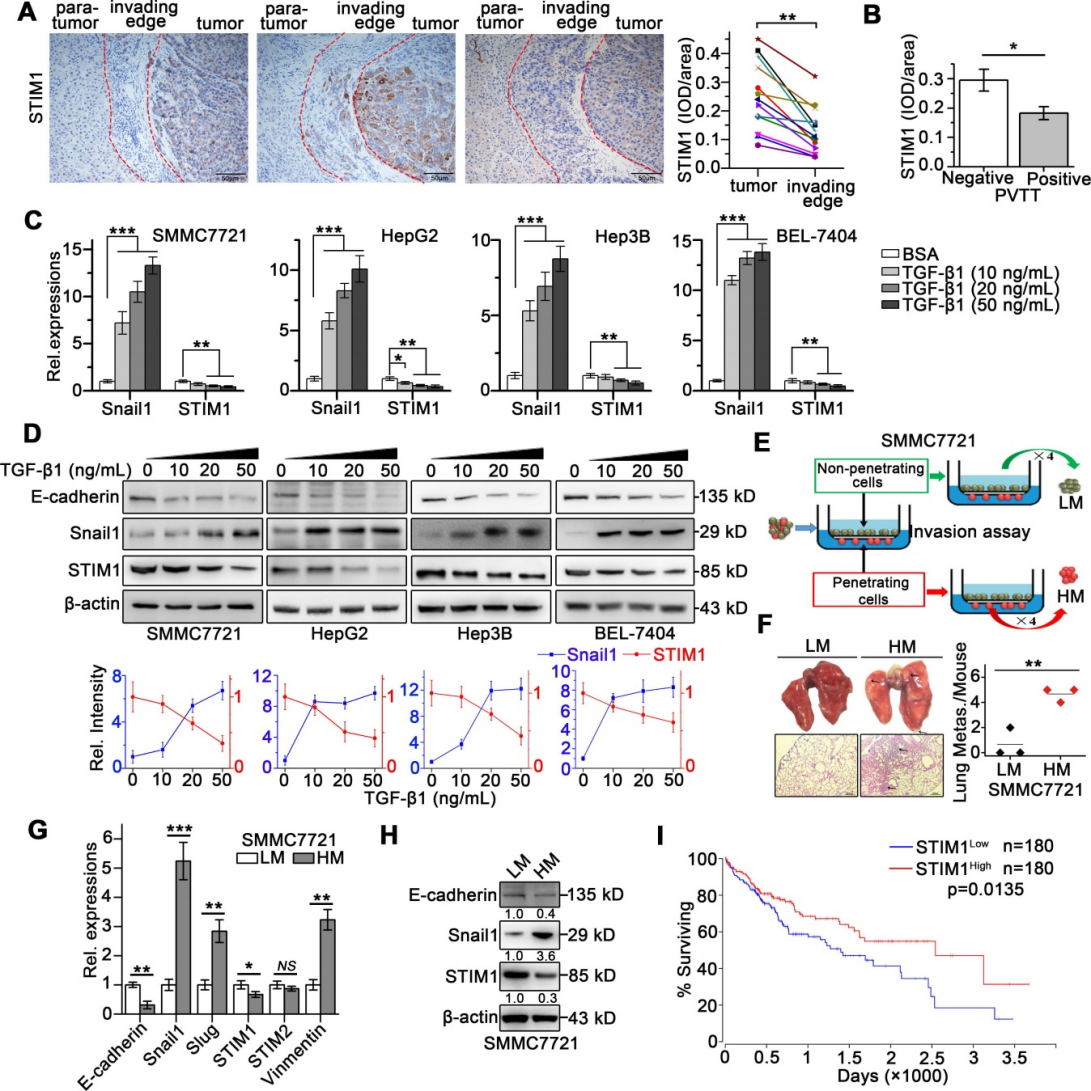
** STIM1 is reduced in tumor invading-edge and metastatic HCC cells.** (**A**) Representative micrographs of STIM1 immunohistochemical analysis (400×) and statistical analysis of integrated optical density (IOD) of STIM1 against immunoglobulin G (IgG) in the invading edge and tumor of 12 HCC patients. (**B**) IOD of STIM1 against IgG in the tumor invading-edge of portal vein tumor thrombus (PVTT)-positive (n = 4) and PVTT-negative (n = 8) HCC samples. (**C**) Snail1 and STIM1 mRNA, (**D**) E-cadherin, Snail1 and STIM1 protein expressions were detected in SMMC7721, HepG2, Hep3B and BEL-7404 treated with TGF-β1 for 48 h. The results were analyzed and normalized against expression with 20 ng/mL bovine serum albumin (BSA) treated cells. (**E**) Diagram that the isolation different metastatic sublines from SMMC7721 cells after 4 rounds of selection, LM: low metastatic, HM: high metastatic. (**F**) Metastatic characteristic of LM- and HM-SMMC7721 sublines in* vivo*, lungs were observed for metastatic nodules on the surface, representative photographs and H&E staining were shown (n = 4 mice per group), arrows point to metastatic nodules. (**G**,** H**) The mRNA (**G**) and protein (**H**) expressions of STIM1, Snail1 and E-cadherin in LM- and HM-SMMC7721 sublines. (**I**) Kaplan-Meier analysis of correlation between the STIM1 expression and overall survival of HCC patients from TGCA (n = 360). Data of (**A**-**D**, **G** and **H**) are expressed as mean ± SEM (n = 3). *p < 0.05, **p <0.01, ***p < 0.001, NS represents no significant difference.

We previously reported that STIM1 is positively correlated with HIF-1α during hypoxic HCC growth 9. Since STIM1 promotes cell migration in lung cancer, breast cancer, and melanoma by regulating focal adhesion turnover 14-17, we speculated that it might also be upregulated in metastatic HCC. However, we found that STIM1 was notably downregulated in the tumor invading-edge (the region between tumor and para-tumor), compared with the corresponding tumor region of the HCC tissue (Figure 1A). Next, we evaluated the STIM1 levels in the tumor invading-edge with/without the portal vein tumor thrombus (PVTT), an essential indicator highly associated with the progression and metastasis of HCC 18, 19. Compared with PVTT negative group, the samples from HCC patients with PVTT showed lower expression of STIM1 in the tumor invading-edge (Figure 1B).

To monitor the dynamic expression of STIM1 during HCC cell invasion and metastasis, we established EMT models of SMMC7721, HepG2, Hep3B, and BEL-7404 cells *via* treatment with transforming growth factor beta 1 (TGF-β1) or under hypoxic condition. We found that TGF-β1 treatment for 48 h significantly enhanced Snail1 expressions, while dramatically repressed STIM1 expression (Figure 1C-D). Under hypoxic condition (1% O_2_), the mRNA and protein levels of STIM1 and HIF-1α were increased at 12 and 24 h; however, they were subsequently reduced at 36 and 48 h. Of interest, Snail1 increased steadily even at 36 and 48 h (Figure S1A-B). We next isolated the sublines with high and low metastatic capacity derived from the SMMC7721 cells (Figure 1E), as previously reported 20, 21. The high metastatic (HM)-sublines displayed higher metastatic activity, while lower proliferating speed, compared with the low metastatic (LM)-sublines (Figure 1F and S2A-E). We found that STIM1 expression was markedly lower in the HM-sublines than in the LM-sublines of SMMC7721 cells (Figure 1G-H). Furthermore, Kaplan-Meier estimates revealed that low STIM1 expression correlated with poor survival among HCC patients *via* microarray data obtained from TCGA database 22 (Figure 1I). These results indicate that STIM1 is down-regulated in metastatic HCC cells compared with proliferating cells, and low STIM1 levels correlated with poor outcomes of HCC patients.

### STIM1 promotes invasion and metastasis as well as anoikis of HCC cells

**Figure 2:**
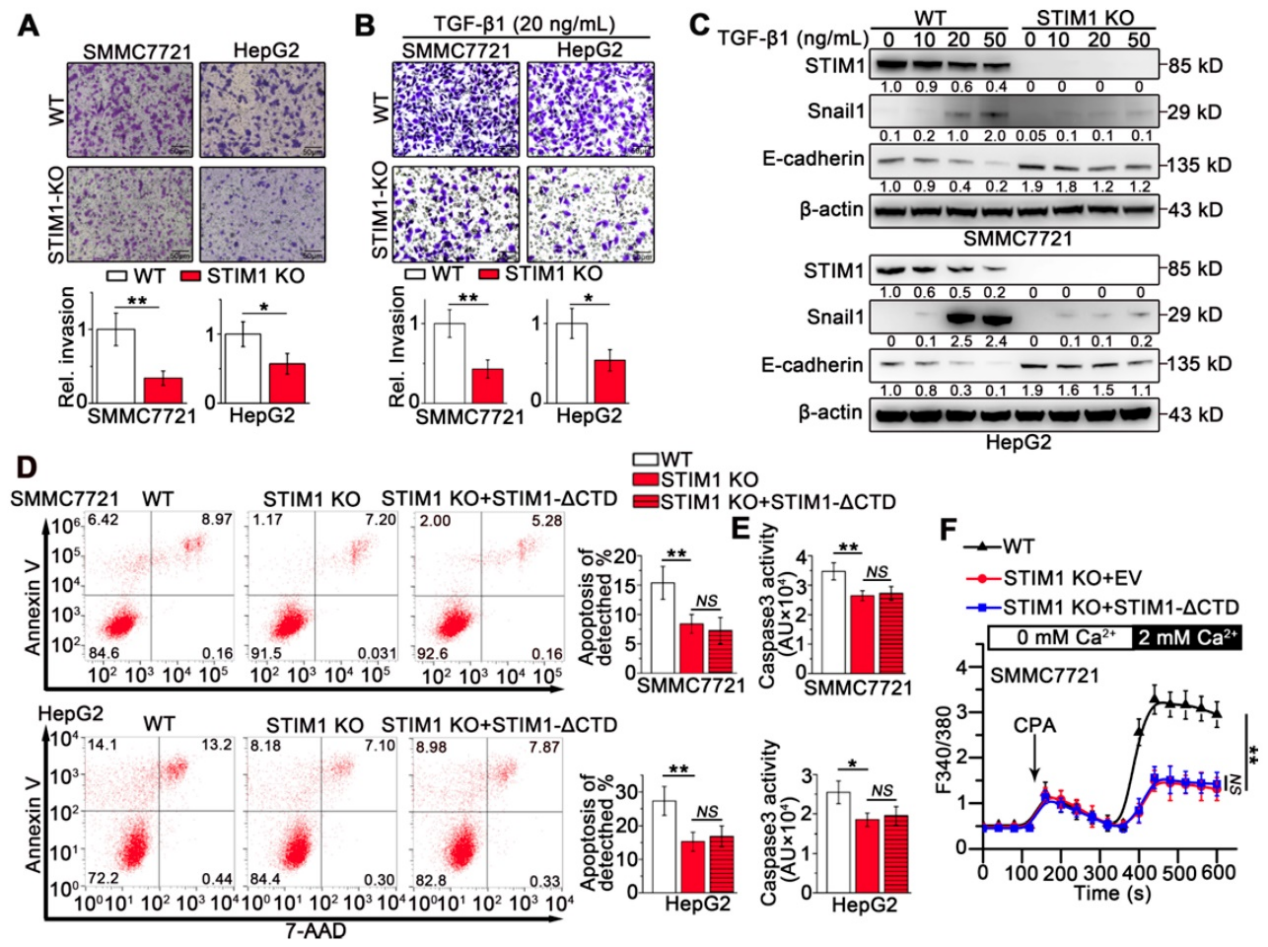
** The effects of STIM1 deficiency on invasion and metastasis in HCCs.** (**A** and **B**) Transwell assays of WT- and STIM1 KO- cells without (**A**) or with TGF-β1 (20 ng/mL) treatment (**B**). (**C**) STIM1, E-cadherin and Snail1 protein levels in WT- and STIM1 KO- cells treated with TGF-β1 for 48 h. (**D**, **E**) Flow cytometry analysis (FACS) (**D**) and caspase 3 activity assay (**E**) were applied to measure the anoikis rate in WT-, STIM1 KO-, STIM1 KO+STIM1-ΔCTD- SMMC7721 cells which were force suspended for 24 h, EV: empty vector; ΔCTD: deletion of the C-terminal domain. (**F**) Ca^2+^ mobilization in WT-, STIM1 KO-, STIM1 KO+STIM1-ΔCTD-SMMC7721 cells, respectively upon cyclopiazonic acid (CPA, 20 mM) stimulation, mean ± SEM of 8 independent cells each group. Data are expressed as mean ± SEM (n = 3). *p < 0.05, **p < 0.01, **p < 0.001, NS represents no significant difference.

To determine the role of STIM1 in the invasion and metastasis of HCC cells, we generated STIM1 knockout (KO)-SMMC7721 and HepG2 cells using a CRISPR/Cas9 system (Figure S3A-D). STIM1 depletion significantly inhibited cell invasion (Figure 2A), clonal formation, and proliferation *in vitro* (Figure S3E-G). Furthermore, both TGF-β1- and hypoxia-induced invasion and metastasis as well as Snail1 expression were blunted when STIM1 was knocked-out in SMMC7721 and HepG2 cells (Figure 2B-C; S4A-B). Of interest, ablation of STIM1 enabled HCC cells to evade anoikis (Figure 2D-E), a programmed cell death triggered through detachment from the substratum 23-25. Moreover, the introduction of a functional mutant* STIM1* with a deletion of the C-terminal domain (STIM1-ΔCTD) failed to reverse anoikis resistance caused by STIM1 deficiency (Figure 2D-F), suggesting that repressed SOCE may contribute to anoikis resistance in STIM1 KO-HCC cells. These data indicate that STIM1 deficiency is required for anoikis resistance in metastatic HCC cells, *i.e.* STIM1 KO contributes to HCC cell survival during metastasis.

### The STIM1-Snail1 negative feedback circuit is involved in HCC pathogenesis and metastasis

**Figure 3:**
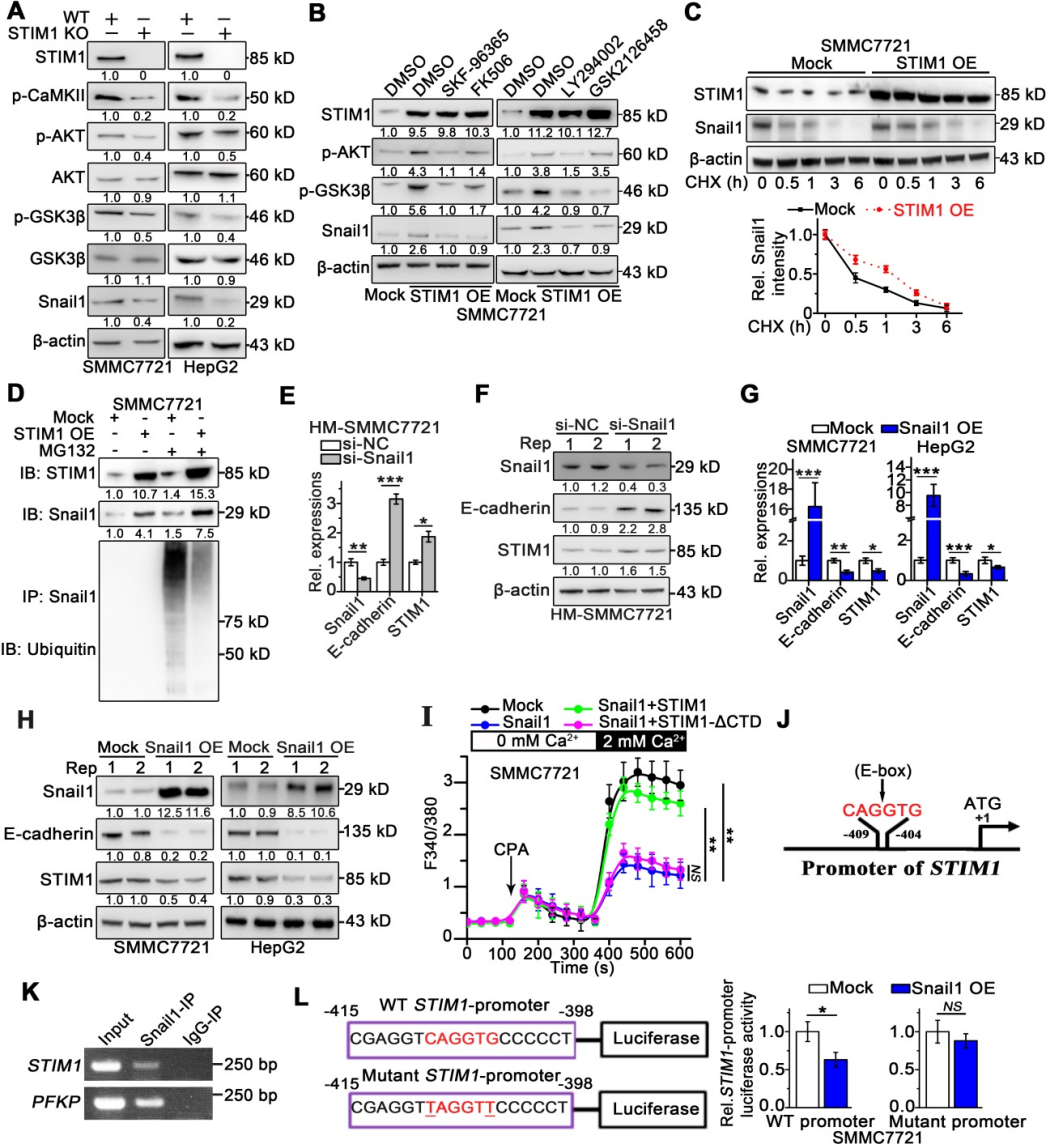
**The interaction between STIM1 and Snail1 in HCC.** (**A**) Indicated protein expressions in WT- and STIM1 KO-SMMC7721 or HepG2 were examined, and β-actin was used as a loading control. (**B**) STIM1 OE-SMMC7721 cells were treated with SKF-96365 (10 µM), FK506 (10 µM), LY294002 (10 µM), GSK2126458 (1 µM) for 24 h; WB was used for measuring STIM1, p-AKT (Thr308), p-GSK-3β (Ser9) and Snail1 protein levels, and β-actin was used as a loading control, DMSO: dimethyl sulfoxide. (**C**) Mock- and STIM1 OE-SMMC7721 cells were treated by cycloheximide (CHX, 1 µM) with different time intervals. Cell extracts were immunoblotted with antibodies against STIM1, Snail1 and β-actin. Snail1 levels (normalized to β-actin) were plotted against CHX treatment durations. (**D**) Mock- and STIM1 OE-SMMC7721 cells were treated with or without MG132 (5 μM). Cell extracts were immunoprecipitated with Snail1 antibody and immunoblotted with antibodies against ubiquitin or Snail1, IP: immunoprecipitation, IB: immunoblotting. (**E**,** F**) HM-SMMC7721 sublines were transfected with scrambled siRNA (si-NC) or si-Snail1, RT-qPCR (**E**) and WB (**F**) to assess STIM1, Snail1 and E-cadherin expressions. (**G**, **H**) RT-qPCR (**G**) and WB (**H**) to assess STIM1, Snail1 and E-cadherin expressions in mock- and Snail1 OE-SMMC7721 and HepG2 cells. (**I**) Ca^2+^ mobilization upon CPA (20 mM) challenge after over-expressing Snail1, Snail1 plus STIM1, Snail1 plus STIM1-ΔCTD in SMMC7721 cells, respectively, mean ± SEM of 8 independent cells. (**J**) Bioinformatics analysis predicted binding site of Snail1 (5'-CAGGTG-3') in the promoter of *STIM1*, black arrow points to transcription start site. (**K**) ChIP assay of Snail1 protein and *STIM1* promoter, representative agarose gel results showing recruitment of Snail1 to the *STIM1* promoter, and *PFKP promoter* used as a positive control. (**L**) Luciferase activity assay of *STIM1* promoter and *STIM1* promoter containing mutant E-box (TAGGTT) in Snail1 OE-SMMC7721. Data are expressed as mean ± SEM (n = 3). *p < 0.05, **p < 0.01, ***p < 0.001, NS represents no significant difference.

Snail1 is essential for the invasion and metastasis of cancer cells, and its stability is regulated by glycogen synthase kinase 3 beta (GSK-3β)-induced ubiquitination 26-28. Since STIM1-mediated SOCE activates the CaMKII/AKT pathway, and AKT also inhibits GSK-3β 10, 29, we speculated that STIM1 might suppress the proteasomal degradation of Snail1 by modulating the SOCE/CaMKII/AKT/GSK-3β pathway. Deficiency of STIM1 significantly reduced Snail1 protein levels and attenuated CaMKII/AKT/GSK-3β signaling (Figure 3A), while it did not influence Snail1 mRNA levels in SMMC7721 and HepG2 cells (Figure S5A). In contrast, STIM1 over-expression (OE) markedly boosted Snail1 protein expression, which was impaired by inhibitors of CaMKII/AKT/GSK-3β signaling pathway (Figure 3B). Thereafter, we assessed Snail1 stability using protein synthesis inhibitor-cycloheximide (CHX), and found that the degradation of Snail1 was significantly inhibited in STIM1 OE-SMMC7721 cells (Figure 3C). Moreover, robust Snail1 ubiquitination after pretreatment with proteasome inhibitor MG132 could be attenuated by over-expression of STIM1 (Figure 3D). Taken together, these data indicate that STIM1 stabilizes and activates Snail1 protein *via* the SOCE/CaMKII/AKT/GSK-3β signaling cascade in HCC cells.

Since the expression patterns of STIM1 and Snail1 differed in metastatic HCC cells, we hypothesized that Snail1 may transcriptionally regulate STIM1 expression. Indeed, knockdown of Snail1 by short interfering RNA (siRNA) upregulated STIM1 expression (Figure 3E-F), as well as led to a moderate promotion of proliferation in HM-SMMC7721 sublines (Figure S5B). On the other hand, over-expression of Snail1 repressed the expressions of STIM1 and E-cadherin in SMMC7721 and HepG2 cells (Figure 3G-H). Nevertheless, the levels of STIM2 and Orai1 were not influenced by over-expressing Snail1 (Figure S5C-D). In addition, ectopic expression of Snail1 attenuated SOCE in SMMC7721 cells. Reintroducing of STIM1 restored SOCE in Snail1 over-expressing cells; however, this was not observed in cells with heterogeneous STIM1-ΔCTD supplementation (Figure 3I). Furthermore, the *in silico* analysis revealed that the *STIM1* proximal promoter harbored a canonical E-box motif (5'-CAGGTG-3') at position -409 to -404 (Figure 3J), which may play an important role in mediating the transcriptional repressor activity of Snail1 30, 31. Chromatin immunoprecipitation (ChIP) analysis validated that DNA fragment of *STIM1* promoter containing the putative Snail1 binding sites could be amplified from the Snail1-immunoprecipitated samples (Figure 3K). Consistently, electrophoretic mobility shift assay (EMSA) revealed that the probe corresponding to the region containing the E-box of the *STIM1* promoter combined with Snail1, and this binding could be abrogated by the unlabeled oligonucleotides (Figure S5E). Moreover, the reporter activity of *STIM1* promoter was suppressed by Snail1 over-expression in SMMC7721, whereas the specific mutation at predicted binding site attenuated the ability of Snail1 to suppress *STIM1* promoter activity (Figure 3L). These results suggest that Snail1 transcriptionally suppresses STIM1 expression *via* binding with the *STIM1* promoter at the E-box motif. Furthermore, Snail1 upregulation accompanied by STIM1 downregulation was observed in the invading-edge of HCC tissues (Figure S5F). Compared with PVTT negative samples, samples from HCC patients with PVTT possessed higher expression of Snail1 at the invading-edge of tumors (Figure S5G). These results indicate that Snail1 transcriptionally suppresses STIM1 expression and represses SOCE during the invasion and metastasis of HCC.

### Restoration of STIM1 abrogates anoikis resistance and metastatic activity of Snail1 OE -HCC cells

**Figure 4:**
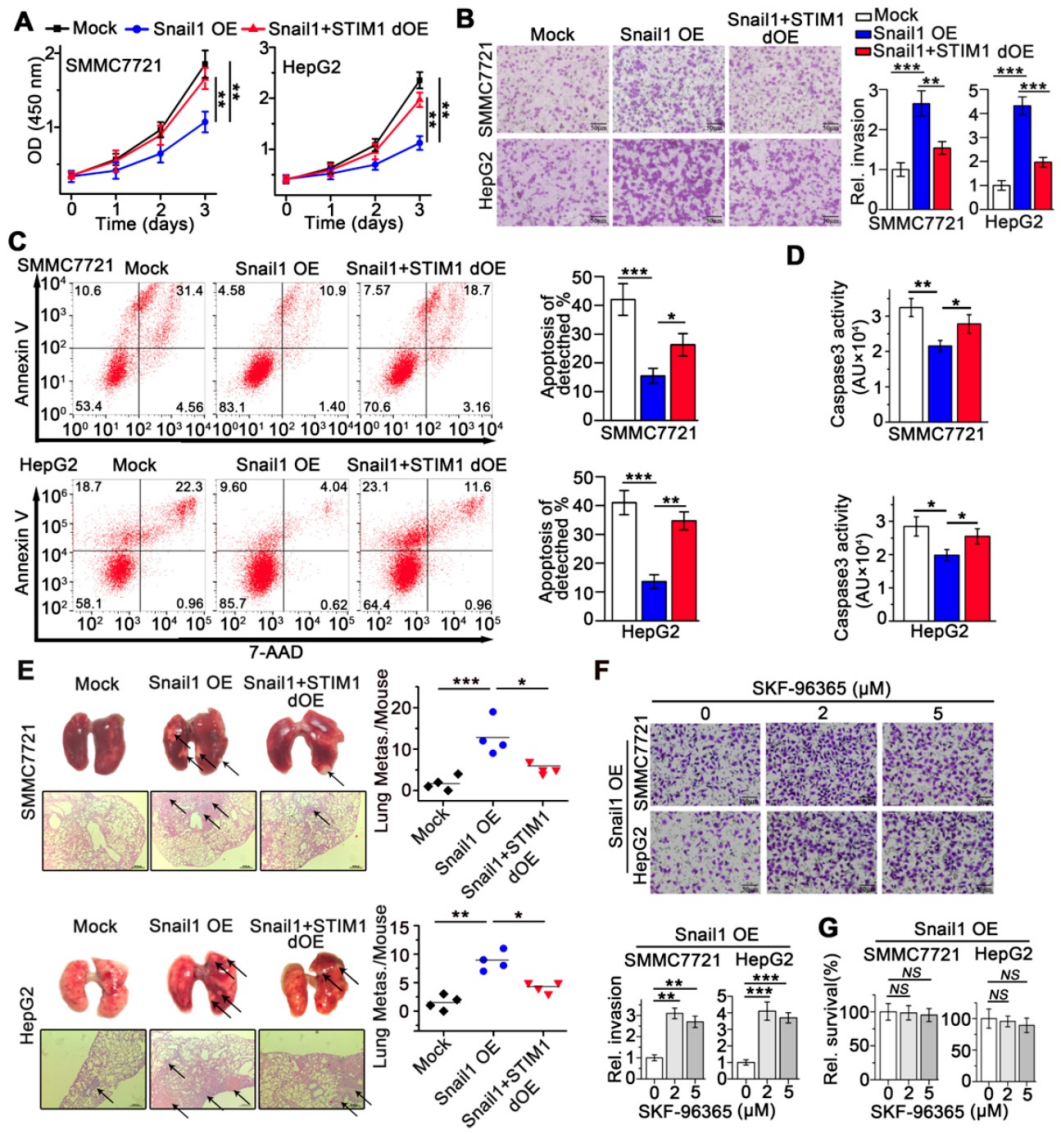
** STIM1 replenish abrogates the anoikis resistance and metastasis of Snail1 OE-cells.** (**A**) Effects of STIM1 on the proliferation of Snail1 OE-SMCC7721 and HepG2 cells. (**B**) Transwell assays for the invasion of WT-, Snail1 OE-, Snail1 plus STIM1 double OE (Snail1+STIM1 dOE)-SMMC7721 and HepG2 cells. (**C**, **D**) FACS (**C**) and caspase 3 activity assay (**D**) were used to measure the anoikis rate in mock-, Snail1 OE- and Snail1+STIM1 dOE-SMMC7721 and HepG2 cells. (**E**) The effects of STIM1 restoration on the metastasis of Snail1 OE-SMMC7721 and HepG2 cells *in vivo*. Lungs were observed for metastatic nodules on the surface, stained by H&E for histological analyses, arrows point to metastatic nodules. Representative photographs and H&E staining were shown (n = 4 mice per group). (**F**) Transwell assays were performed to detect the effects of different concentrations SKF-96365 on the invasion ability of Snail1 OE-SMMC7721 and HepG2 cells. (**G**) CCK-8 assay was applied to examine the effects of SKF-96365 with different concentrations on the survival of Snail1 OE-SMMC7721 and HepG2 cells. Data of (**A-D, F** and** G**) are expressed as mean ± SEM (n = 3). **p < 0.01, ***p < 0.001, ***p < 0.001, NS represents no significant difference.

We next investigated the effects of STIM1 on the metastatic activity of Snail1 OE-HCC cells. Interestingly, STIM1 restoration facilitated cell proliferation (Figure 4A), but attenuated anoikis-resistance and metastasis of Snail1 OE-SMMC7721 and HepG2 cells both *in vitro* and *in vivo* (Figure 4B-E). Furthermore, low concentrations of SKF-96365, a specific SOCE inhibitor, enhanced the invasive capability of Snail1 OE-HCC cells (Figure 4F-G). These results further indicate that repressed STIM1/SOCE is required for avoiding anoikis and promoting the invasion and metastasis of Snail1 OE-HCC cells.

### STIM1 deficiency in HCC cells leads to decreased glycolysis and enhanced fatty acid oxidation

**Figure 5:**
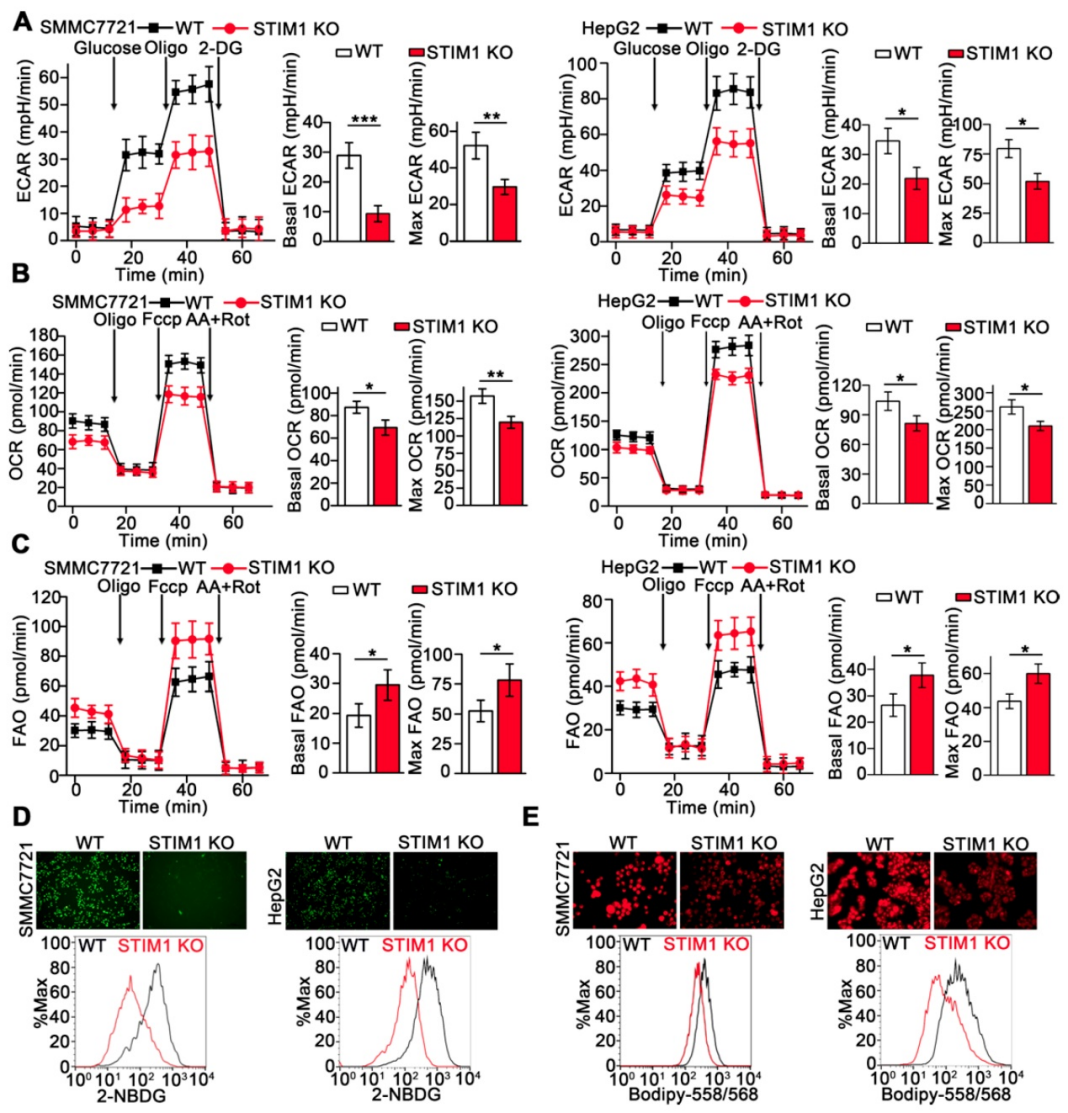
** STIM1 deficiency rewires aerobic glycolysis towards FAO.** (**A-C**) ECAR (**A**), OCR (**B**) and FAO (**C**) caused by STIM1 deficiency in SMMC7721 and HepG2 cells were measured by Seahorse XF24 analyzer. Oligo: Oligomycin, 2-DG: 2-Deoxy-D-glucose, Fccp: Carbonyl cyanide 4-(trifluoromethoxy) phenylhydrazone, AA/Rot: Antimycin A/Rotenone. (**D**, **E**) The glucose uptake (**D**) and intracellular lipid content (**E**) in WT- and STIM1 KO-SMMC7721 or HepG2 cells were determined by fluorescence microscope and FACS. Data are expressed as mean ± SEM (n = 3). *p < 0.05, **p < 0.01, ***p < 0.001.

Metabolic reprogramming is fundamental for orchestrating the proliferation and metastasis of tumor cells 32-35. We examined the metabolic phenotype resulting from STIM1 deficiency in HCC cells using Seahorse XFp cellular flux analyzer. Loss of STIM1 reduced the glycolysis-driven extracellular acidification rate (ECAR) and oxygen consumption rate (OCR) but enhanced fatty acid oxidation (FAO) in SMMC7721 and HepG2 cells (Figure 5A-C). Moreover, deletion of STIM1 dramatically decreased glucose uptake and intracellular lipid deposition in SMMC7721 and HepG2 cells (Figure 5D and E).

**Figure 6:**
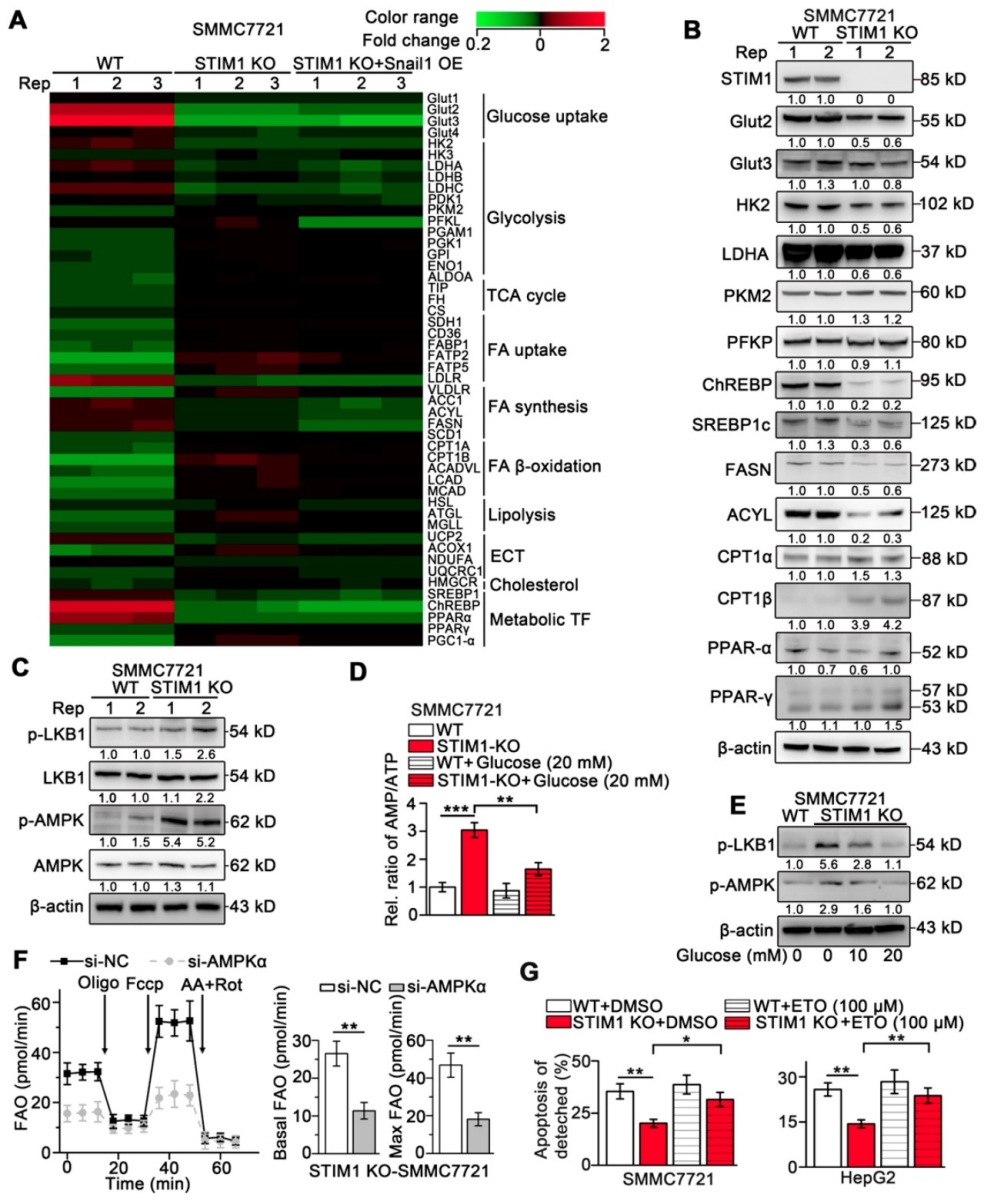
** Lacking of STIM1 rewires aerobic glycolysis towards AMPK-activated FAO.** (**A**) PCR-array was applied to examine the expression changes of key metabolic genes caused by STIM1 KO and STIM1 KO+Snail1 OE (GSE148129). (**B**) Protein levels of indicated metabolic molecules in WT- and STIM1 KO-SMMC7721 cells. (**C**) Protein levels of LKB1/AMPK pathway in WT- and STIM1 KO-SMMC7721 cells. (**D**) The AMP/ATP ratio in WT- and STIM1 KO-SMMC7721 with or without glucose (20 mM). (**E**) Effects of glucose on the expressions of p-LKB (Ser428) and p-AMPK (Thr172) in STIM1 KO-SMMC7721 cells. (**F**) FAO in STIM1 KO-SMMC7721 cells transfected with si-NC or si-AMPKα. (**G**) Effects of ETO (100 μM) on the anoikis of STIM1 KO-SMMC7721 and HepG2 cells were examined by FACS, as well as their corresponding WT-group. ETO: etomoxir. Data are expressed as mean ± SEM (n = 3). *p < 0.05, **p < 0.01, ***p < 0.001.

Our and other earlier studies have established that HIF-1 promotes glycolysis and *de novo* lipogenesis and inhibits FAO in HCC, and STIM1-mediated SOCE stabilizes HIF-1α 9, 36-38. To elucidate the mechanism underlying STIM1-mediated regulation of glucose and lipid metabolism, we performed a PCR-array (GSE148129). We observed that genes involved in glucose uptake including glucose transporter 2 (*GLUT2*) and *GLUT3* and glycolytic genes including hexokinases (*HK2* and *HK3*), lactate dehydrogenase A (*LDHA*), and pyruvate dehydrogenase kinase 1 (*PDK1*) were significantly downregulated. Genes in fatty acid synthesis (FAS) including acetyl-coA carboxylase 1 (*ACC1*), fatty acid synthase (*FASN*), and ATP citrate lyase (*ACLY*) were markedly downregulated, while the key enzymes involved in FAO including carnitine palmitoyl-transferase A (*CPT1A*) and long-chain acyl-CoA dehydrogenases (*LCAD*) were upregulated in STIM1 KO-SMMC7721 cells (Figure 6A). Of note, *GLUT2*, *HK2*, *PDK1*, *ACYL*, *FASN*, and *LCAD* are direct targets of HIF-1 39, 40. Besides, the Snail1 protein expression was reduced in STIM1 KO-HCC cells, while Snail1 restoration couldn't reverse the expression changes of metabolic enzymes triggered by STIM1 deletion (Figure 6A), suggesting that the switched metabolism from aerobic glycolysis towards FAO after depletion of STIM1 is in a Snail1-independent manner. Consistently, the protein levels of Glut2, HK2, LDHA, ChREBP, SREBP1c, FASN and ACYL were dramatically decreased, while CPT1α and CPT1β were markedly increased in STIM1-deficient HCC cells (Figure 6B). The correlation between STIM1 and metabolism-related genes was analyzed using microarray data of 238 HCC patients obtained from Gene Expression Omnibus (GEO), which showed that STIM1 positively correlated with several genes involved in glycolysis and FAS, but negatively correlated with FAO (Figure S6A).

In addition, UK-5099, an inhibitor of mitochondrial pyruvate intake, but not BPTES (a glutaminase inhibitor), suppressed OCR in STIM1 KO-HCC cells (Figure S6B), indicating that decreased glycolysis is implicated in reduced OCR resulting from the ablation of STIM1. Furthermore, SOCE inhibitor SKF-96365 suppressed ECAR and OCR, but enhanced FAO in SMMC7721 cells (Figure S6C-E). Supplementation of STIM1-ΔCTD did not reverse the expression changes of metabolic enzymes upon STIM1 KO (Figure S6F). These results demonstrate that STIM1 promotes glycolysis and FAS during HCC pathogenesis *via* activating SOCE.

To elucidate the mechanism of enhanced FAO in STIM1-deficient HCC cells, we examined the key regulatory factors of FAO. Deletion of STIM1 activated phospho-liver kinase B1 (p-LKB1) and phospho-adenosine monophosphate-activated protein kinase (p-AMPK), but did not influence expressions of PPAR-α and PPAR-γ in SMMC7721 cells (Figure 6B-C). AMPK, a critical sensor of cellular energy in response to energy stress, is activated by CaMKII or LKB1 41. Activated AMPK inactivates ACC1* via* Ser79 phosphorylation, which leads to a reduction in malonyl-CoA synthesis, thereby promoting FAO by alleviating the inhibition on CPT1 42, 43. Because the CaMKII pathway was blocked in STIM1 KO-HCC cells, AMPK might be activated by glucose deficiency. Indeed, we found that the ratio of adenosine monophosphate (AMP) and adenosine triphosphate (ATP) was elevated by STIM1 knockout (Figure 6D). Glucose supplementation not only reduced the AMP/ATP ratio, but also decreased the levels of p-LKB1 and downstream active p-AMPK (Figure 6D-E). Furthermore, AMPKα knockdown markedly attenuated STIM1 deficiency-triggered FAO (Figure 6F). Etomoxir (ETO), a specific CPT1 inhibitor blocking the FAO pathway, significantly promoted the apoptosis of the detached STIM1 KO-HCC cells, suggesting that elevated FAO contributed to anoikis resistance in STIM1-deficient HCC cells (Figure 6G). These findings demonstrate that STIM1 deficiency attenuates the glycolysis and FAS pathway, while activates the LKB1/AMPK-dependent FAO pathway in HCC cells.

### Catabolic FAO triggered by STIM1 deficiency is required for Snail1-driven invasion and metastasis

**Figure 7:**
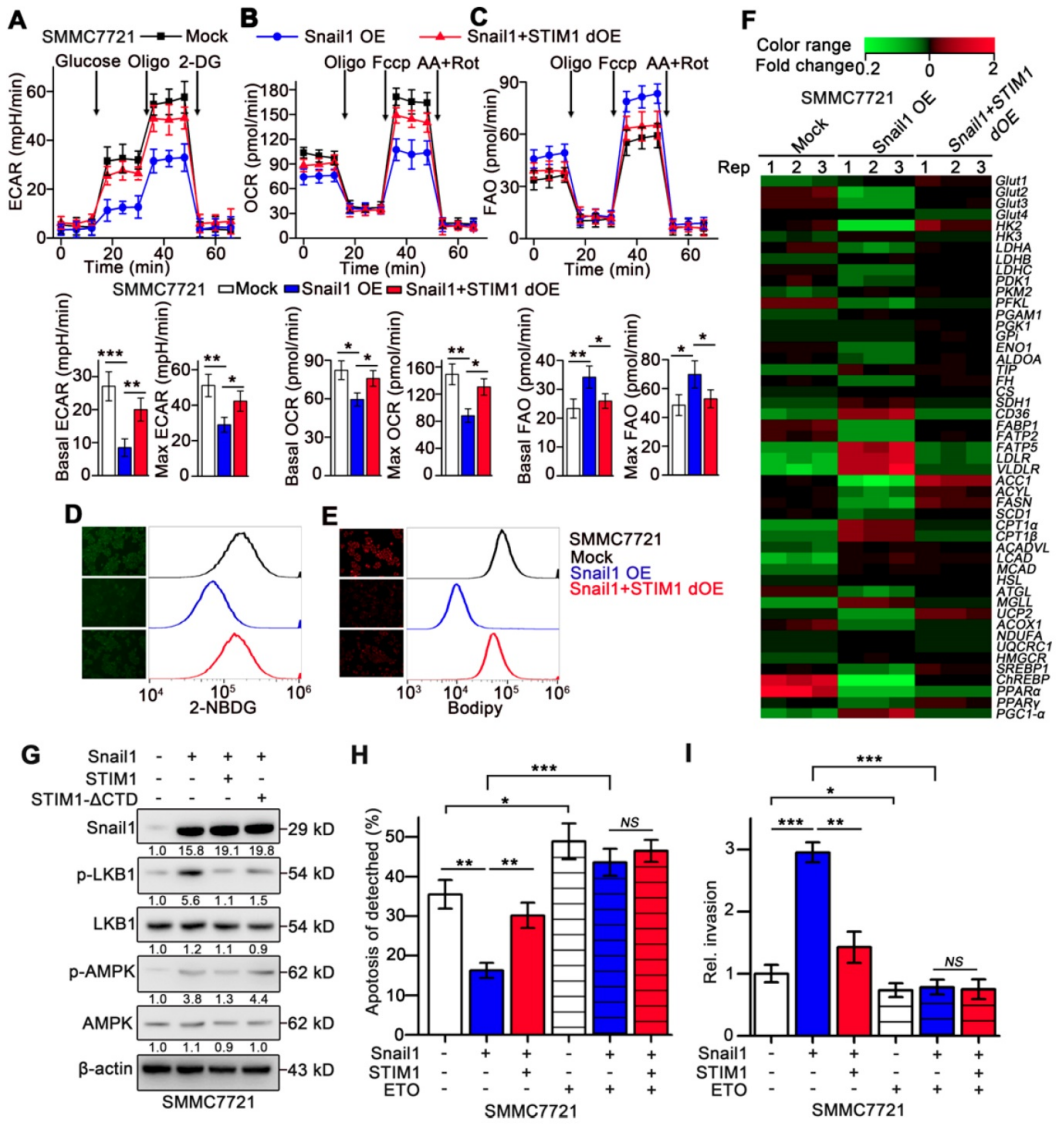
** Metabolic switch triggered by Snail1 could be reversed by STIM1 restoration.** (**A**-**C**) ECAR (**A**), OCR (**B**) and FAO (**C**) in mock-, Snail1 OE-, Snail1+STIM1 dOE-SMMC7721 cells. (**D**,** E**) Glucose uptake (**D**) and intracellular lipid deposition (**E**) in mock-, Snail1 OE- and Snail1+STIM1 dOE-SMMC7721 cells. (**F**) PCR-array was applied to examine the expression of key metabolic genes in mock, Snail1 OE, Snail1 plus STIM1 dOE SMMC7721 cells (GSE135901). (**G**) Effects of STIM1 and STIM1-ΔCTD on the LKB1/AMPK pathway in Snail1 OE-SMMC7721 cells. (**H**) Effects of ETO (100 μM) on anoikis of mock-, Snail1 OE- and Snail1+STIM1 dOE-SMMC7721 cells were examined by FACS. (**I**) Effects of ETO (100 μM) on the invasion ability of mock-, Snail1 OE- and Snail1+STIM1 dOE-SMMC7721 cells *via* transwell assays. Data are expressed as mean ± SEM (n = 3). *p < 0.05, **p < 0.01, ***p < 0.001. NS represents no significant difference.

Metastatic cells exhibit enhanced catabolism but decreased anabolism 4, 5, 44, we speculated whether STIM1 deficiency contributed to the anabolism/catabolism switch during the EMT of HCC cells. Treatment with TGF-β1 repressed ECAR and moderately accelerated FAO in SMMC7721 cells (Figure S7A-B). Similarly, Snail1 OE-HCC cells exhibited significantly impaired OCR and ECAR but enhanced FAO, compared with the mock group. These metabolic changes caused by Snail1 OE were largely reversed upon STIM1 supplementation (Figure 7A-C). Furthermore, glucose uptake and lipid deposition were significantly reduced in Snail1 OE-HCC cells, which could be obviously eliminated by STIM1 supplementation (Figure 7D-E). Consistently, Snail1 OE inhibited the expressions of multiple genes related to glycolysis and FAS, while upregulated several genes involved in lipid uptake, lipolysis and FAO pathways (GSE135901) (Figure 7F). Supplementation of STIM1 also could remove the expression changes of metabolic enzymes caused by Snail1 OE (Figure 7F). Moreover, STIM1, but not STIM1-ΔCTD, could reverse the trend of the LKB1/AMPK pathway activated by Snail1 OE, validating that STIM1 downregulation triggered FAO in Snail1 OE-HCC cells (Figure 7G). In addition, when FAO pathway was blocked by ETO, STIM1 restoration could not alleviate the anoikis resistance or invasion activity driven by Snail1 (Figure 7H-I). These results indicate that STIM1 deficiency contributes to the metabolic switch from glycolysis and FAS to FAO, which is required for the invasion and metastasis driven by Snail1 in HCC cells.

## Discussion

STIM1-mediated SOCE contributes to cell migration in various tumors, including breast cancer, gastric cancer, colorectal cancer, and melanoma, by modulating focal adhesion turnover and myosin II contraction 14-17. Unexpectedly, our data showed that STIM1 was downregulated in the invading-edge in comparison with the corresponding tumor tissue of HCC, which correlated with PVTT formation and poor prognosis of HCC patients. STIM1-activated SOCE promoted Snail1 expression through the CaMKII/AKT/GSK-3β pathway during proliferation. Of interest, Snail1 in turn transcriptionally suppressed STIM1 by binding with *STIM1* promoter during EMT. The intracellular Ca^2+^ promotes anoikis, thus inhibiting extracellular Ca^2+^ influx, which is essential for the survival of metastatic cancer cells 45. We found that repressed STIM1/SOCE was required for preventing anoikis during metastasis. Thus, STIM1 expression is temporally and differentially regulated during EMT, which orchestrates HCC pathogenesis and metastasis.

Although Snail1 directly activates the transcription of several genes, it is extensively considered as a transcriptional repressor 30, 31, 46, 47. The repressor activity of Snail1 is dependent on binding to canonical E-box motif (5'-CACCTG-3' or 5'-CAGGTG-3') in the promoter of target gene *via* Cys2-His2 zinc-fnger domain 30, 48. In addition, a proposed Snail1-responsive motif (5'-TCACA-3') has been identified in the promoters of several genes activated by Snail1 including *ZEB1*, *MMP9*, and *p15INK4* 31, 46. In this study, bioinformatics analysis revealed that the *STIM1* proximal promoter (-1005 ~ +1 from the transcription starting site) harbored a canonical Snail1-binding E-box, whereas no motif of (5'-TCACA-3') was found. Moreover, ChIP and EMSA analysis confirmed that Snail1 could bind to the E-box in *STIM1* promoter. The *STIM1*-promoter activity was dramatically suppressed by Snail1 over-expression, while the activity of E-box mutated *STIM1*-promoter was not altered. Therefore, our results indicate that Snail1 transcriptionally suppresses STIM1 expression by binding to the E-box of *STIM1* promoter in HCC cells.

Disordered Ca^2+^ signal plays a critical role in regulating metabolism during tumor progression 49-51. On one hand, cytosolic Ca^2+^ orchestrates the activity of the Ca^2+^-dependent metabolic enzymes 10, 52-55. For instance, the activity of OXPHOS crucially depends on Ca^2+^-dependent a-ketoglutarate- and isocitrate-dehydrogenases in the mitochondrial matrix 53, 54; and pyruvate dehydrogenase (PDH) is phosphorylated and inactivated at ser293 by the Ca^2+^-dependent phosphatase- pyruvate dehydrogenase kinases (PDK), thereby OXPHOS is switched towards aerobic glycolysis 52. On the other hand, cytosolic Ca^2+^ signaling can indirectly activate various metabolic transcription factors, including nuclear factor of activated T cells (NFAT), AP-1 transcription factor (AP-1), and cAMP-response element-binding protein (CREB) 10, 11, 56. Our data showed that both STIM1 KO and SOCE inhibitors-SKF-96365 suppressed glycolysis, but enhanced FAO in HCC cells, indicating that Ca^2+^ signal regulated by STIM1/SOCE is involved in metabolic regulation of HCC.

Metabolic reprogramming is necessary to maintain rapid growth and metastasis of cancer cells 4, 57, 58. Our results showed that deletion of STIM1 significantly inhibited cell proliferation in HCC cells. Notably, knockout of STIM1 also markedly downregulated several downstream targets of HIF-1, including *GLUT2*, *HK2*, *PDK1*, *ACYL* and *FASN*, which are key enzymes for glycolysis and FAS, respectively. Literature have shown that HCC cells exhibit a high rate of glucose-derived *de novo* FAS to fulfill the biosynthesis of membranes and signaling molecules 57, 59, 60. Conversely, the reduced lipid consumption (FAO) in cancer cells is expected to sustain uncontrolled rapid proliferation. For instance, HIF-1-mediated suppression of FAO through downregulation of MCAD and LCAD is critical for the growth of HCC cells 36, CD147-mediated inhibition of FAO is beneficial for HCC growth and metastasis 61. However, metastatic cancer cells undergo metabolic stress, which are primarily characterized by glucose deficiency 4, 62. The metabolic switch from anabolism (glycolysis and FAS) to catabolism (FAO) is required for protecting cells against starvation and anoikis 25, 63, 64. The present results suggest that STIM1 is a key regulator of metabolism to maintain a balance between anabolism and catabolism. During proliferation, STIM1 promotes glycolysis and FAS but suppresses FAO, thus promoting HCC cell proliferation and Snail1 expression. However, with the upregulation of Snail1, the attenuation of STIM1-mediated SOCE results in the reduction of FAS but the acceleration of FAO, which subsequently inhibits cell proliferation and induces anoikis resistance and metastasis in HCC cells.

In conclusion, our results reveal that STIM1 is a metabolic checkpoint that orchestrates the invasion and metastasis in HCC by switching aerobic glycolysis to FAO. STIM1 repression is required for the 'metabolic switch' from anabolic to catabolic metabolism in HCC cells undergoing EMT, suggesting that temporal targeting the STIM1-Snail1 signaling circuit is a potential therapeutic alternative for metastatic HCC.

## Materials and Methods

### Human samples

Paraffin-embedded primary hepatocarcinoma tissues were obtained from patients at Xinqiao Hospital (Chongqing, China). Clinicopathological characteristics of HCC patients were summarized in Table S1. The use of clinical specimens in this study was approved by the Xinqiao Hospital ethics committee of the Amry Medical University.

### Cell lines

SMMC7721, HepG2, Hep3B and HEK293T cell lines were purchased from the American Type Culture Collection (ATCC, Rockville, MD, USA). BEL-7404 were obtained from the Cell Bank of Type Culture Collection of Chinese Academy of Sciences (Shanghai, China). All cell lines had been authenticated and tested for *Mycoplasma*, and all cells were maintained according to the manufacturer's instructions and passages <10 were used in this study.

### Establishment of high and low metastatic sublines of SMMC7721

A pair of SMMC7721 subpopulations with differently metastatic ability were established according to previously studies 20, 21, 65. Transwells (BD biosciences, San Jose, CA) with 8-μm pore size filters covered with Matrigel (BD biosciences) inserted into 6-well plates were used to build an *in vitro* invasion model. In brief, SMMC7721 cells (60-70% confluent) were serum starved for 24 h before they were digested and suspended in medium without fetal bovine serum (FBS). Cell density was adjusted to 5×10^5^ cells/mL and 1 mL cell suspension was added into chamber pre-incubated with 0.5 mL DMEM without FBS. The lower chamber was added with DMEM with 20% (v/v) FBS. After 24 h incubation at 37 °C and 5% CO_2_, cells from upside (U) and downside (D) of the chamber membrane were harvested and cultured, respectively. In the subsequent three rounds of selection, only the upside cells derived from the first-generation of U-subpopulation and the membrane penetrated cells from the original D-sublines were obtained. After four rounds of continual separation, we acquired one pairs of SMMC7721 cell sublines, which were named as high metastatic (HM)- and low metastatic (LM)-sublines, respectively.

### Gene expression

Total RNA was extracted using Trizol (TAKARA, Japan) and reversely transcribed using PrimeScript™ RT reagent Kit with gDNA Eraser (TAKARA). mRNA expression was assessed by Real-time quantitative polymerase chain reaction (RT-qPCR) using TB Green® Premix Ex Taq™ II (TAKARA) on BioRad CFX384 (Bio-Rad, CA) with 40 cycles at 95 °C for 10 s, 59 °C for 20 s and 72 °C for 30 s. Gene expression levels were analyzed using the delta Ct method and normalized by subtracting that of control β-actin mRNA. The gene-specific primers used in RT-qPCR experiments were listed in Table S2.

### Western blotting

For HCC cells, whole lysates were prepared by direct lysis in RIPA buffer with PMSF (Beyotime, Beijing, China) and phosphatase inhibitors (Cwbiotech, Beijing, China). Protein concentration was quantified using BCA Protein Assay Kit (Beyotime) and 40 μg total protein/well was loaded. Samples were then separated by 4-12% Bis-Tris PAGE electrophoresis and transferred to PVDF membrane for detection. Western blots were probed overnight at 4 °C with specific primary antibodies in Tris-Buffered Saline Tween-20 (TBST) containing 5% skim milk. After washed for 3 times with TBST, the membranes were incubated for 1 h at room temperature with a respective IgG-HRP labled second antibody (1:5,000) in TBST containing 5% skim milk. Antigens were revealed using a chemiluminescence assay (Pierce, Rockford, USA). Quantification of bands was achieved by densitometry using the FluorChem HD2 system (ProteinSimple, Santa Clare, CA, USA). The antibodies used in WB analysis were listed in Table S3.

### H&E staining and Immunohistochemistry

The tissue specimens were fixed at least 24 h in 10% neutral-buffered formaldehyde immediately after surgical removal, and then dehydrated in isopropyl alcohol, followed by clearing of alcohol by xylene. Subsequently, the dehydrated specimens were embedded in paraffin, standard staining with hematoxylin and eosin (H&E) was performed. For immunohistochemistry (IHC), tumor sections were deparaffinized, then incubated in citrate buffer (pH 6.0) at 95 °C for 45 min for antigen retrieval. Next, the specimens were blocked with 5% goat serum for 30 min, which followed by incubating with the primary antibodies rabbit immunoglobulin G (IgG, 1:200), STIM1 (1:100) or Snail1 (1:100) respectively overnight at 4 °C. After three washes, tissue sections were incubated with HRP anti-rabbit IgG (1:200) at room temperature for 60 min and followed by incubated with DAB solution and then counterstained with haematoxylin. Staining results were captured by an ortho microscope (Olympus, Tokyo, Japan) under high-magnification (400×). After that, the integrated optical density (IOD) of STIM1, Snail1 and IgG in the tumor invading-edge and corresponding tumor region were measured using ImageJ software (Media Cybernetics, Bethesda, MD, USA), and the mean density (IOD/area) of STIM1 and Snail1 against IgG in different areas of cancer specimens were calculated by ImageJ software. The antibodies used in IHC were listed in Table S3.

### Animal studies

Male BABL/c nude mice (5~6 weeks old) obtained from the Charles River (Beijing, China) were used for *in vivo* metastasis assay and subcutaneous xenograft, randomization was conducted. The use of experimental animals was based on the National Institutes of Health (NIH) guidelines. For the lung metastatic model, 2×10^6^ HCC cells were injected into the blood of nude mice through tail vein. After 6 weeks, the mice were sacrificed and metastatic organs (lung) were excised and the micro-metastases were examined using a dissecting microscope. The metastasis was confirmed by H&E staining.

### Inhibitors, recombinant proteins and other reagents

SKF-96365 (HY-100001), FK506 (HY-13756), MG132 (HY-13259), Cycloheximide (HY-12320), BPTES (HY-12683), UK-5099 (HY-15475), Etomoxir (HY-50202), Puromycin (HY-B1743) and Blasticidin S (HY-103401A) were obtained from MedChemExpres (Monmouth Junction, NJ, USA). LY29402 (LY294002) and GSK2126458 (S2658) were purchased from Selleckchem (Houston, TX, USA). Glucose (A2494001), Lipofectamine 2000 (11668027), 2-NBDG (N13195), Bodipy 558/568 (D3835) and Fura 2-AM (F1221) were obtained from Thermo Fisher Scientific (Waltham, MA, USA). Poly-2-hydroxyethyl methacrylate (poly-HEMA, 529257), dimethyl sulfoxide (DMSO, 34869) and cyclopiazonic acid (239805) were purchased from Sigma-Aldrich (St. Louis, MO, USA). Recombinant TGF-β1 (240-B-002) was obtained from R&D Systems, Inc. (Minneapolis, MN, USA).

### Seahorse XFp metabolic flux assays

The rate of metabolic flux was determined by seahorse XFp extracellular flux analyzer (Agilent Technologies, Santa Clara, CA, USA). 5,000 cells/well were seeded in Seahorse XFp cell culture plates and allowed to adhere overnight. Then the medium was replaced with substrate-limited medium for 16 h. **For ECAR detection**: 60 min before the examination, cells were washed twice and replaced with Seahorse XF DMEM Medium (pH 7.4), then the cell plate was incubated in CO_2_ free incubator at 37 °C for 1 h, the EACR was detected according to Glycolysis Stress Test protocols. The following concentrations for each drug were used during ECAR acquisitions: Glucose, 10 mM; Oligomycin (Oligo), 1 μM; 2-Deoxy-D-glucose (2-DG), 50 mM. The rates of ECAR were normalized to protein levels in each well. **For FAO detection:** 60 min before the assay, cells were washed twice and replaced with FAO assay medium containing 111 mM NaCl, 4.7 mM KCl, 1.25 mM CaCl_2_, 2 mM MgSO_4_, 1.2 mM NaH_2_PO_4_, supplemented with 2.5 mM Glucose, 0.5 mM carnitine and 5 mM HEPES at a final pH of 7.4. Palmitate-BSA was applied at a final concentration of 0.1 mM just before the start of the assay. The FAO was detected according to XFp Cell Mito Stress Test protocols. The following concentrations for each drug were used during FAO acquisitions: Oligo, 4 μM; Fccp, 2 μM; Antimycin A/Rotenone (AA/Rot), 2 μM. The rates of FAO were normalized to protein levels in each well. **For OCR detection:** 60 min before the assay, cells were washed twice and replaced with Seahorse XF DMEM Medium (pH 7.4) containing 10 mM Glucose, 1 mM pyruvate and 2 mM Glutamine. The OCR was detected according to XFp Cell Mito Stress Test protocols. The following concentrations for each drug were used during OCR acquisitions: Oligo, 4 μM; Fccp, 2 μM; AA/Rot, 2 μM. The rates of OCR were normalized to protein levels in each well.

### Glucose uptake and intracellular lipid content measurement

**Glucose uptake** was analyzed directly using the fluorescent glucose analog 2-NBDG. HCC cells were incubated in glucose-free RPMI medium containing 100 mM 2-NBDG for 90 min at 37 °C in dark, and the amount of 2-NBDG taken up by cells was assessed by flow cytometry analysis (FACS). For fluorescence examine, HCC cells grown on coverslips were fixed with 4% paraformaldehyde for 10 min, then cells were washed 3 times in cold PBS and stained with 100 mM 2-NBDG for 90 min at 37 °C in dark, and cell images were photographed using a fluorescence microscope (Leica, Wetzlar, Germany). **For lipid content measurement**, 2×10^5^ cultured HCC cells were incubated in the presence of 1 μM fluorescent lipid probe Bodipy 558/568 for 30 min at 37 °C in dark. Then the labeled cells were washed and re-suspended in cold PBS and the lipid content was quantified using FACS as mentioned above. For fluorescence examine, HCC cell lines grown on coverslips were fixed with 4% paraformaldehyde for 10 min. Following fixation, cells were washed 3 times in cold PBS and stained with 1 μM Bodipy 558/568 for 30 min at 37 °C in dark, and cell images were photographed using a fluorescence microscope (Leica).

### Plasmids, lentiviral and siRNA

The recombinant plasmids containing human *STIM1* and *SNAI1* were purchased from GeneCopoeia (Rockville, MD, USA), *STIM1*-ΔCTD mutant recombinant plasmid which containing 1-440 AA of *STIM1* was synthesized and inserted into pReceiver-Lv197 lentiviral vector. Lentivirus was produced in HEK293T cells according to the instruction manual of Lenti-Pac™ HIV Expression Packaging Kit (GeneCopoeia). Viral supernatant was harvested at 48~72 h post-transfection, passed through a 0.45 µm polyethersulfone low protein-binding filter, diluted 1:2 (v/v) with fresh medium containing polybrene (7.5 mg/mL) and used to infect the target cells at 80% confluence. Three days after infection, blasticidin S (10 μg/mL) or puromycin (3 μg/mL) was used to select the cells with stable expression of lentivirus. Over-expression efficiency of STIM1 or Snail1 was evaluated by immunoblotting and RT-qPCR. Besides, siRNAs targeting human Snail (SIGS0002558-1), AMPKα (SIGS0004655-4) were obtained from RiboBio (Guangzhou, China).

### Calcium imaging

Calcium imaging was carried out as previously described 9. In brief, cells were placed on coverslips coated with poly-D-lysine. Intracellular Ca^2+^ was monitored using the fluorescent Ca^2+^ indicator Fura 2-AM according to the manufacture's instruction. Images were collected at 6-second intervals. Measurements of intracellular Ca^2+^ concentration ([Ca^2+^]i) of single cells were performed using an inverted fluorescence microscope (Nikon, Japan). The standard extracellular solution contained (mM): 140 NaCl, 5 KCl, 2 CaCl_2_, 1 MgCl_2_·6H_2_O, 10 HEPES, 10 Glucose, pH 7.4. Ca^2+^-free extracellular solution was prepared by replacing CaCl_2_ with equimolar amounts of MgCl_2_ and 0.5 mM EGTA was added. After loading, cells were washed three times in the above solution and then left for 15 min to allow for further de-esterification. Background fluorescence signals were collected at the same rate for the same wavelengths (340 and 380 nm) and were subtracted from the corresponding fluorescence images. The results (∆F/F0) were expressed as ratios of fluorescence signals measured at 340 nm to fluorescence signals measured at 380 nm during a response divided by the ratio obtained in resting conditions (that is, before the addition of an agent). ∆F/F0 was used to assess the amplitude of [Ca^2+^]i in these cells.

### CRISPR/Cas9 targeted deletion of *STIM1*

To knock out *STIM1* gene, we designed single guided RNA (sgRNA) sequences (Forward 5′-CAC CGC ATC ATC GTC CAT CAG TTT G-3′; Reverse: 5′-AAA CCA AAC TGA TGG ACG ATG ATG C-3′) for human *STIM1* gene and cloned the targeting sequences into the lentiCRISPR v2 vector (Addgene, Watertown, MA, USA). Lentivirus for *STIM1* sgRNA, vector control were generated in HEK293T cells by standard methods using lenti-packaging vectors. SMMC7721 and HepG2 cells were then infected with the lentivirus for 48 h and selected with puromycin (3 μg/mL) for 10 days, then established the monoclonal cells. STIM1 deletion in individual monoclonal cell line was further verified by DNA sequencing and WB.

### Chromatin immunoprecipitation PCR

Chromatin immunoprecipitation (ChIP) assays were performed using a ChIP Kit (Thermo Fisher Scientific) as described previously 9. Briefly, chromatin from cells was crosslinked with 1% formaldehyde for 10 min at room temperature, sheared to an average size of 500 bp, and immunoprecipitated with Snail1 antibody (CST, 3879) and IgG. The ChIP-PCR primers of* STIM1* (forward: 5'-AGC TTC TGC TGC TCG CCG CTC TTC-3'; reverse: 5'-GGA CCC ACT GTT GGA CCT GAG GAG-3') were designed to amplify the promoter region containing the putative Snail1-binding site (5'-CAGGTG-3') at the *STIM1* promoter. Using *PFPK* as a positive control for ChIP analysis, The ChIP-PCR primers of *PFKP* (forward: 5'- CTA GAG CCC CCA ACC AGA GT-3'; reverse: 5'- GTG TGG GCA GGA GCA TCT AC -3') were designed according to the previously published study 66. Each immunoprecipitated DNA sample was amplified using PCR and ChIP-PCR products were detected by agarose gel electrophoresis.

### Electrophoretic mobility shift assay (EMSA)

EMSA was performed using the LightShift^®^ Chemiluminescent EMSA Kit (Thermo Scientific, USA) according to the manufacturer's instructions. Biotin-labeled probe containing the E-box region acquired from the promoter of the *STIM1* gene (-435 ~ -383) was synthesized by Sangon Biotech (Shanghai, China). DNA binding reactions were performed in 20 μL system containing 4.5 μL protein product was mixed with 1 μL (1 pmol) of the labeled probe, 2 µL 10×binding buffer, 1 µL 50% Glycerol, 100 mM MgCl_2_, 1 µg Poly (dI • dC), 100 mM MgCl_2_ and 200 mM EDTA. Reaction products were separated by electrophoresis in 6% polyacrylamide gels containing 0.5% ×TBE. Thereafter, the protein-DNA complexes were transfered onto a positively charged nylon membrane (Millipore, USA) and detected by chemiluminescence. Additional unlabeled oligonucleotides were used as competitor at 100-fold molar excess.

### *STIM1* promoter luciferase assay

To analyze the* STIM1* promoter activity, the promoter region (-1005 ~ +1 from the transcription starting site) was synthesized by GenScript co., LTD (Nanjing, China) and subcloned into pGL3-basic vector (Promega, Madison, WI, USA), and E-box sequence 5'-CAGGTG was mutated to 5'-AAGGTA. To examine the *STIM1* promoter activity, the mock- and Snail1 OE-cells were transfected with 1 μg of reporter vector and 20 ng of pSV-Renilla expression vector. Luciferase and Renilla activities were measured using the dual-luciferase reporter system kit (Promega), and the luciferase activity was normalized with renilla activity. The results were expressed as the averages of the ratios of the reporter activities from triplicate experiments.

### Co-immunoprecipitation for ubiquitin assay

Mock- and STIM1 OE-SMMC7721 cells had been grown on 10-cm dishes, and were treated with 10 μM MG132 for an additional 4 h before harvested in lysis buffer with PMSF. The supernatants collected from centrifugation were pre-clarified by the protein A/G PLUS-agarose (Santa Cruz Biotechnology, Dallas, TX) overnight at 4 °C, followed by immunoprecipitation with antibody against Snail1 (CST , 3879) for 6 h at 4 °C, then washed five times with the cold lysis buffer containing PMSF, mixed with adequate amount of 1×SDS buffer and heated denaturation. Followed by immunoblotting analysis for ubiquitin and Snail1.

### ATP and AMP assay

ATP concentrations were tested with enhanced ATP assay kit obtained from Beyotime according to the manusfactuer's protocol. Cells were lysed with ATP lysis-buffer and centrifuged at 1.5×10^4^ g for 10 min at 4 °C. The supernatants were collected and stored on ice. Before ATP test, 100 µL of ATP working solution was added to 1.5 mL tube and incubated for 5 min at room temperature. Next the supernatant were transferred to 100 µL of ATP working solution, mixed quickly, and the amount of luminescence emitted was immediately measured with Varioskan Flash (Thermo Fisher). The luminescence data were normalized against those sample protein amounts. AMP concentrations were tested with AMP-Glo assay kit obtained from Promega (USA) according to the manusfactuer's protocol. Upon completion of the enzyme reaction, the first step, requiring addition of AMP-Glo Reagent I, depleted the remaining ATP (for ATP requiring enzyme reactions, e.g.) and converted the AMP generated during the enzyme reaction to ADP. This step was completed in a 60 min incubation. As several AMP generating enzymatic reactions are dependent on ATP or cAMP as substrate and generate pyrophosphate (PPi) as a product in addition to AMP, which is a potent luciferase inhibitor. In the second step, AMP Detection Solution was added, which was concomitantly detected by a luciferase/luciferin system with Varioskan Flash (Thermo Fisher Scientific).

### Anoikis assay and caspase 3 activity determination

Poly-2-hydroxyethyl methacrylate (poly-HEMA, Sigma-Aldrich) was prepared by dissolving it in 95% ethanol (v/v) to a concentration of 12 mg/mL and subsequently added to cell culture wells at a density of 5 mg/cm^2^. Cells were cultured for 24 h using poly (HEMA)-treated (suspended) dishes. Then, anoikis rate of cells was determined by the FITC Annexin V/7-AAD Apoptosis Kit (BD Biosciences, San Jose, CA, USA) and analyzed by BD FACS Calibur system, and data were analyzed with FlowJo software (San Carlos, CA, USA). Caspase 3 activity were tested by Caspase 3 Activity Assay Kit obtained from Beyotime according to the manusfactuer's protocol. Cell lysates were prepared by incubating 2×10^6^ cells/mL in extraction buffer for 30 min on ice. Lysates were centrifuged at 13,000×g for 15 min, and the supernatants were collected. The protein concentrations were determined by BCA protein assay (Beyotime). Cellular extracts (40 μg) were then incubated in a 96-well microtitre plate with 20 ng Ac-DEVD-pNA for 2 h at 37 °C. Caspase 3 activity was measured by cleavage of the Ac-DEVD-pNA or Ac-LEVD-pNA substrate to pNA, the absorbance of which was measured by Varioskan Flash (Thermo Fisher Scientific) at 405 nm. Relative caspase activity was calculated as a ratio of emission of treated cells to untreated cells.

### Cell proliferation and viability

Cell proliferation and viability at the indicated incubation time were determined by Cell Counting Kit-8 (CCK-8) assay (Dojindo, Japan) according to the manusfactuer's protocol, the data were quantified with Varioskan Flash (Thermo Fisher Scientific) at 450 nm.

### *In vitro* migration and invasion assays

For wound-healing migration assays, a single scratch wound was created by dragging a 10 μL plastic pipette tip across the cell surface. The area of a defined region within the scratch was measured using ImageJ software. The extent to which the wound had closed over 24 h was calculated and expressed as a percentage of the difference between time 0 and 24 h. For invasion assays, Transwells (BD biosciences, CA) with 8-μm pore size filters covered with matrigel (BD biosciences) were inserted into 24-well plates. The cells were serum-starved overnight and then added in the upper chamber (3×10^4^ cells per-insert) and the culture medium supplemented with 20% FBS was used as a chemoattractant in the lower chamber. After incubation for 24 h, non-invading cells that remained on the upper surface of the filter were removed, and the cells that had passed through the filter and attached to the bottom of the membrane were fixed in methanol and stained with 0.2% crystal violet. Numbers of the invasive cells in seven randomly selected fields from triplicate chambers were counted in each experiment under a phase-contrast microscope.

### Statistical analysis

Overall survival of HCC patients was calculated using the Kaplan-Meier method, data were available online (http://www.oncolnc.org/) 22, and the differences in survival curves were analyzed using the log-rank test. Statistical analysis was performed using the statistical program Origin 9.1 (OriginLab, Northampton, MA, USA). All data were presented as mean ± SEM and were analyzed by Student's t test or one-way ANOVA. *P* values < 0.05 were considered statistically significant. Heatmaps were presented for up- and down-regulated genes using the Heatmap illustrator (version 1.0.3.7).

### Data and materials availability

The accession numbers for the microarray published here are GEO: GSE148129 and GSE135901.

## Supplementary Material

Supplementary figures and tables.

## Abbreviations

2-DG: 2-Deoxy-D-glucose
AA/Rot: Antimycin A/Rotenone
ACC1: acetyl-coA carboxylase 1
ACLY: ATP citrate lyase
AMP: adenosine monophosphate
AMPK: adenosine monophosphate-activated protein kinase
AP-1: AP-1 transcription factor
ATP: adenosine triphosphate
BSA: bovine serum albumin
Ca^2+^: calcium
CaMKII: Calcium/Calmodulin-dependent protein kinase-II
ChIP: chromatin immunoprecipitation
CHX: cycloheximide
CPT1: palmitoyl-transferase 1
CREB: cyclic adenosine monophosphate response element-binding protein
DMSO: dimethyl sulfoxide
ECAR: glycolysis-driven extracellular acidification rate
EMT: epithelial-mesenchymal transition
EMSA: electrophoretic mobility shift assay
ER: endoplasmic reticulum
ETO: etomoxir
FACS: flow cytometry analysis
FAO: fatty acid oxidation
FAS: fatty acid synthesis
FASN: fatty acid synthase
Fccp: carbonyl cyanide 4-(trifluoromethoxy) phenylhydrazone
GEO: Gene Expression Omnibus
GLUT: glucose transporter
GSK-3β: glycogen synthase kinase 3 beta
HCC: hepatocellular carcinoma
H&E: hematoxylin and eosin
HIF-1α: hypoxia-inducible factor-1 alpha
HK: hexokinases
HM: high metastatic
IHC: immunohistochemistry
IOD: integrated optical density
LCAD: long-chain acyl-CoA dehydrogenases
LDHA: lactate dehydrogenase A
LKB1: liver kinase B1
LM: low metastatic
KO: knockout
NFAT: nuclear factor of activated T cells
OE: over-expression
OCR: oxygen consumption rate
Oligo: oligomycin
OXPHOS: oxidative phosphorylation
O_2_: oxygen
PDK1: pyruvate dehydrogenase kinase 1
PDH: pyruvate dehydrogenase
PI: propidium iodide
PPAR: peroxisome proliferator activated receptor
PVTT: portal vein tumor thrombus
RT-qPCR: real-time quantitative polymerase chain reaction
siRNA: short interfering RNA
SOCE: store-operated Ca^2+^ entry
STIM1: stromal-interaction molecule 1
sgRNA: single guided RNA
TGF-β1: transforming growth factor-beta 1
WB: western blotting
WT: wild type
ΔCTD: deletion of the C-terminal domain
