# STIM1 is a metabolic checkpoint regulating the invasion and metastasis of hepatocellular carcinoma: Erratum

**DOI:** 10.7150/thno.119395

**Published:** 2025-06-15

**Authors:** Huakan Zhao, Guifang Yan, Lu Zheng, Yu Zhou, Halei Sheng, Lei Wu, Qi Zhang, Juan Lei, Jiangang Zhang, Rong Xin, Lu Jiang, Xiao Zhang, Yu Chen, Jingchun Wang, Yanquan Xu, Dingshan Li, Yongsheng Li

**Affiliations:** 1 Clinical Medicine Research Center, Xinqiao Hospital, Army Medical University, Chongqing 400037, China.; 2 Institute of Cancer, Xinqiao Hospital, Army Medical University, Chongqing 400037, China.; 3 Department of Hepatobiliary Surgery, Xinqiao Hospital, Army Medical University, Chongqing 400037, China.

The authors sincerely apologize for the inadvertent use of incorrect representative images in our previously published paper. This error occurred during the assembly of the figures by the first author and specifically pertains to the transwell images in Figures 2B and 4B. The corrected figures are presented below. We affirm that these corrections do not alter the results or conclusions of our paper. The authors express their sincere apologies to the Journal and its readers for any confusion this may have caused.

## Figures and Tables

**Figure A:**
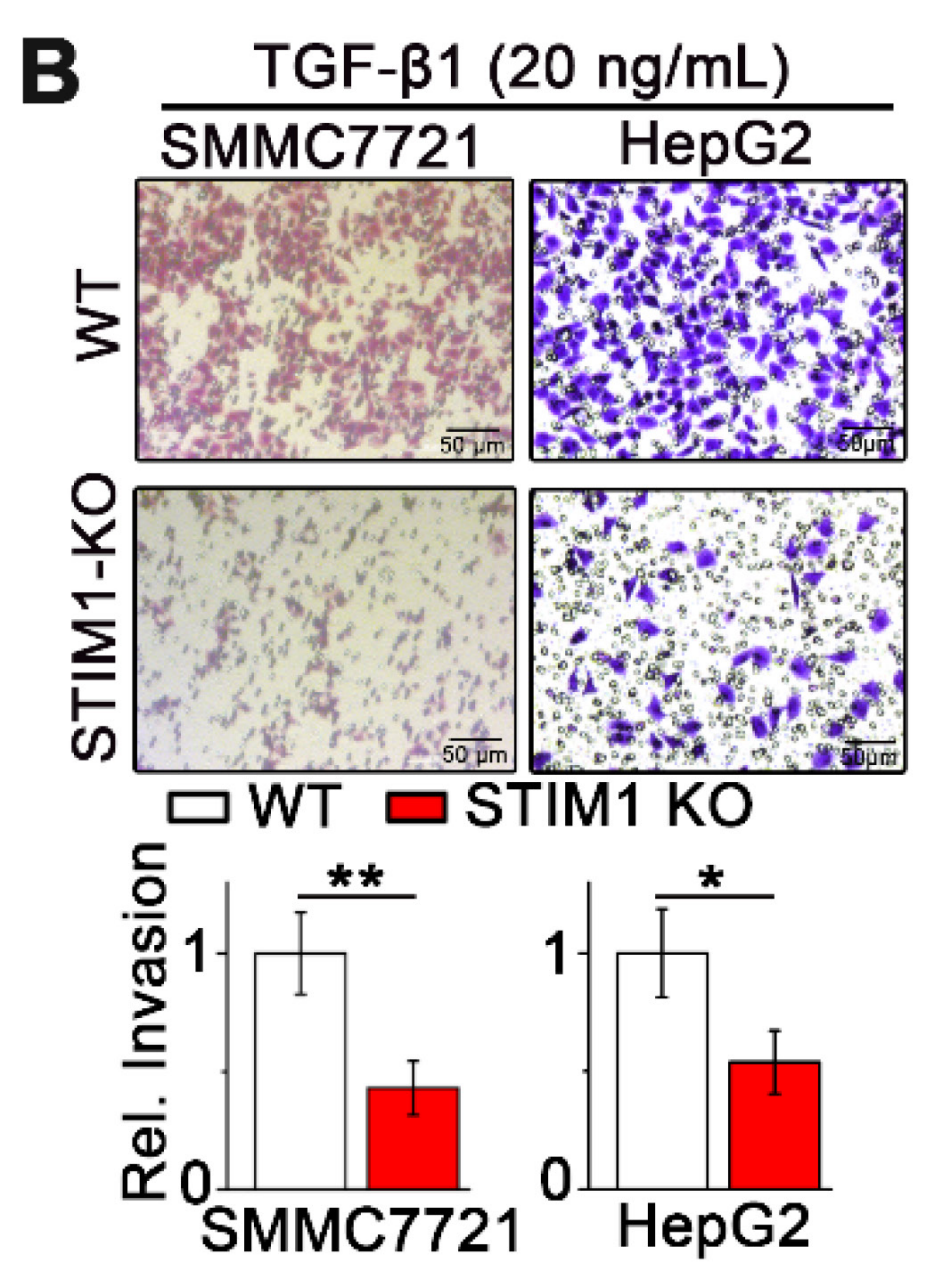
** Corrected Figure 2B** Transwell assays of wild-type (WT) and STIM1 knockout (KO) SMMC7721 and HepG2 cells subjected to treatment with TGF-β1 (20 ng/mL).

**Figure B:**
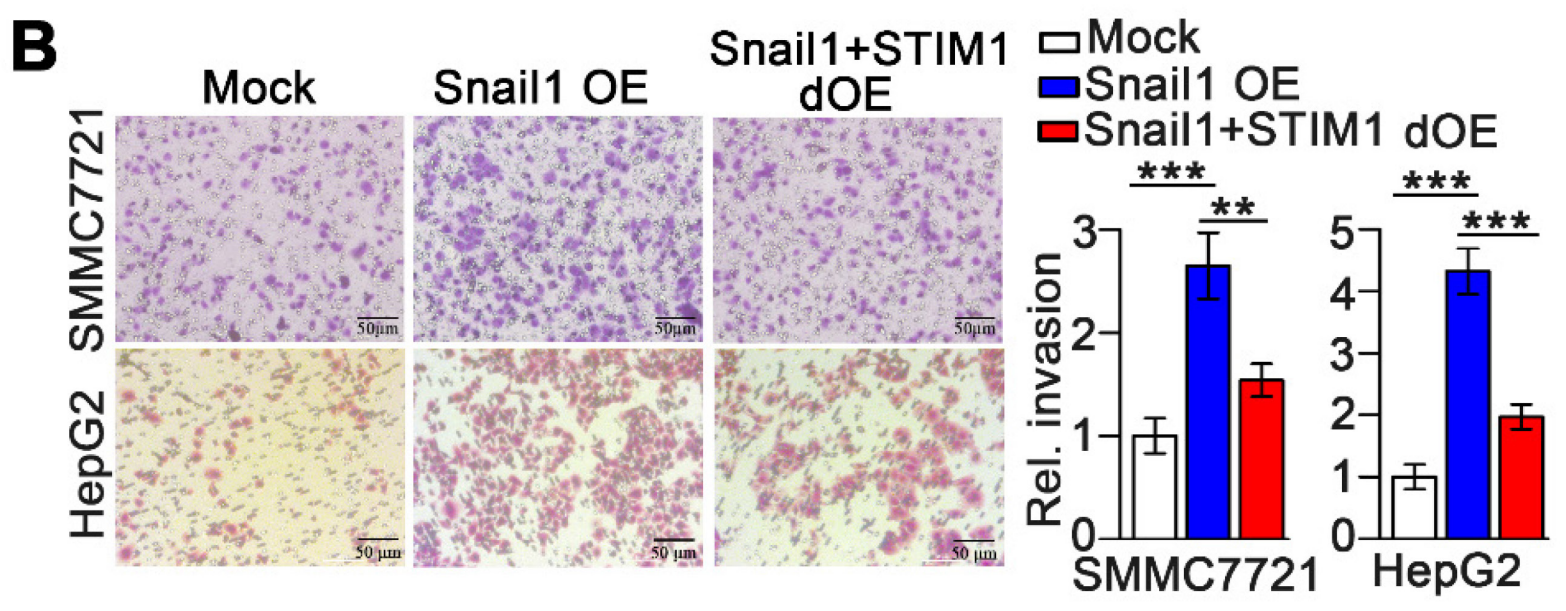
** Corrected Figure 4B** Transwell assays evaluating the invasion of WT, Snail1 overexpressing (OE), and Snail1 plus STIM1 double overexpressing (Snail1+STIM1 dOE) SMMC7721 and HepG2 cells.

