# Supplementary material for: STIM1 is a metabolic checkpoint regulating the invasion and metastasis of hepatocellular carcinoma
Source: Theranostics. 2020 May 16;10(14):6483–99. doi: 10.7150/thno.44025 (PMC7255033; doi:10.7150/thno.44025)
Supplement: Supplementary file 1 — Supplementary figures and tables. [file thnov10p6483s1.pdf]

Supplementary Materials

Supplementary Figures

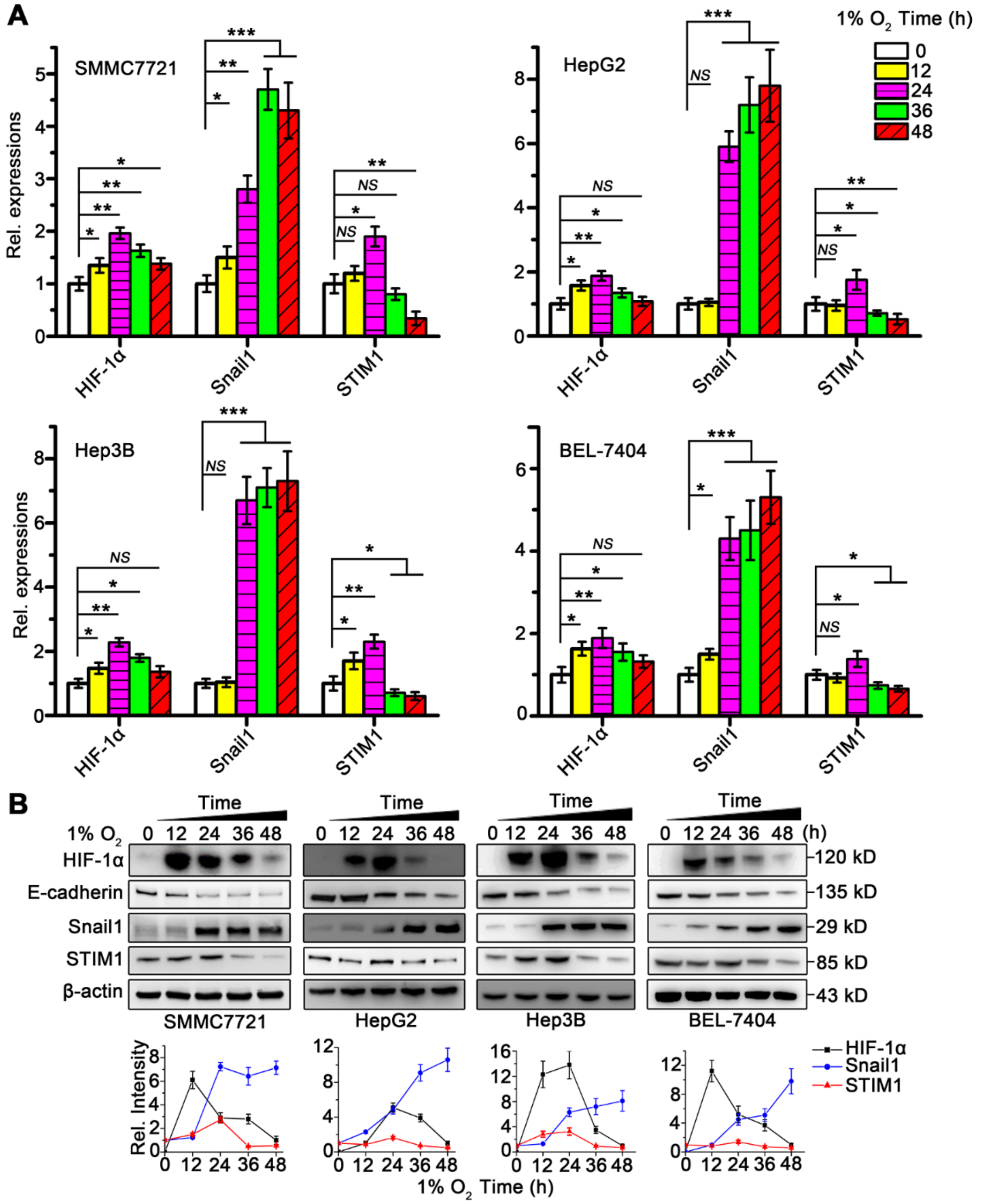

Figure S1. The expressions of STIM1, HIF-1α and Snail1 are temporally and differentially regulated in HCC cells under hypoxia treatment.

(**A**) The mRNA levels of HIF-1α, Snail1 and STIM1, (**B**) the protein expressions of HIF-1α, E-cadherin, Snail1 and STIM1 were detected in SMMC7721, HepG2, Hep3B and BEL-7404 cells cultured in hypoxic condition (1% O_2) with different time intervals. The results were analyzed and normalized against the expression under normoxia. Data are expressed as mean ± SEM (n = 3). *$p < 0.05$, **$p < 0.01$, ***$p < 0.001$, NS represents no significant difference.

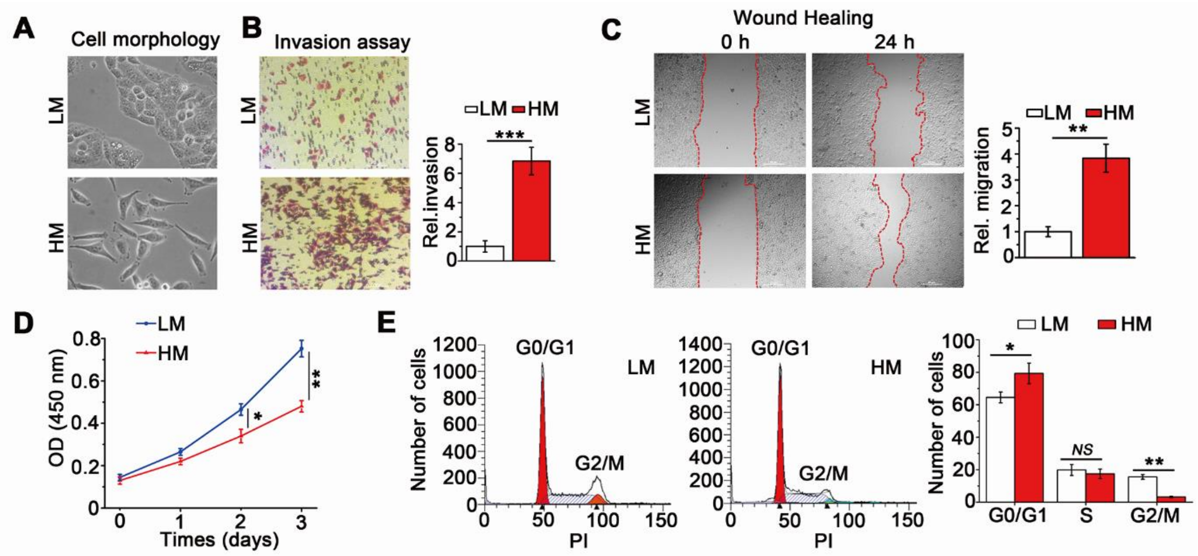

Figure S2. HM-SMMC7721 subline displays higher metastatic and lower proliferative activity.

(**A**–**C**) The morphology (**A**), transwell assay (**B**) and wound-healing assay (**C**) were applied to evaluate the metastatic activities of HM- and LM-SMMC7721 sublines. (**D**) CCK-8 assay was used to examine proliferation. (**E**) Propidium iodide (PI)-staining/FACS was performed to determine the cell cycle progression of HM- and LM-SMMC7721 sublines. Data are expressed as mean ± SEM (n = 3). *$p < 0.05$, **$p < 0.01$, ***$p < 0.001$, NS represents no significant difference.

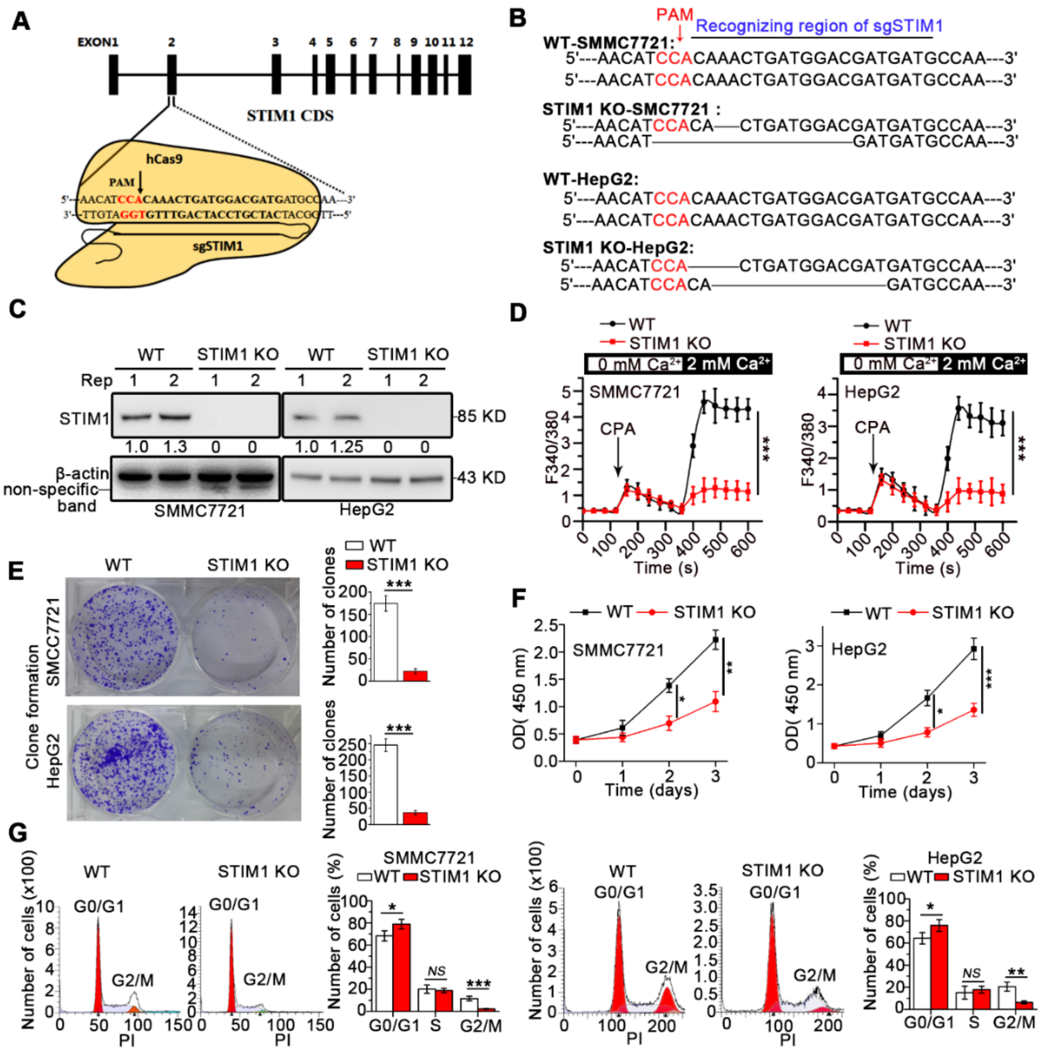

Figure S3. Knockout of STIM1 dampens the proliferation of HCC cells.

(**A**) The Diagram depicts that the nuclease hCas9 recruited by a single guided RNA (sgRNA) specifically recognizing a region spanning the STIM1 codon (sgSTIM1) cleaves the *STIM1* gene. The vertical arrow showed the potential cleavage site. PAM: protospacer adjacent motif. (**B**) Sequences of the Cas-9-edited-genome region in STIM1 knockout (KO)-SMMC7721 and HepG2 monoclones were analyzed by DNA sequencing, WT: wild type. (**C**) STIM1 expressions in STIM1 KO-SMMC7721 and HepG2 monoclones were measured by WB. (**D**) Ca^{2+} mobilization in WT- and STIM1 KO-cells upon cyclopiazonic acid (CPA, 20 mM) challenge. The intracellular Ca^{2+} concentration was expressed as mean ± SEM of 8 independent cells each group. (**E-G**) STIM1 knockout inhibited proliferation in SMMC7721 cells. Clone formation assay (**E**), CCK-8 assay (**F**) and PI-staining/FACS measuring cell-cycle (**G**) were used to evaluate for the proliferative characteristics of STIM1 KO-cells. Data are expressed as mean ± SEM (n = 3). *$p < 0.05$, **$p < 0.01$, ***$p < 0.001$, NS represents no significant difference.

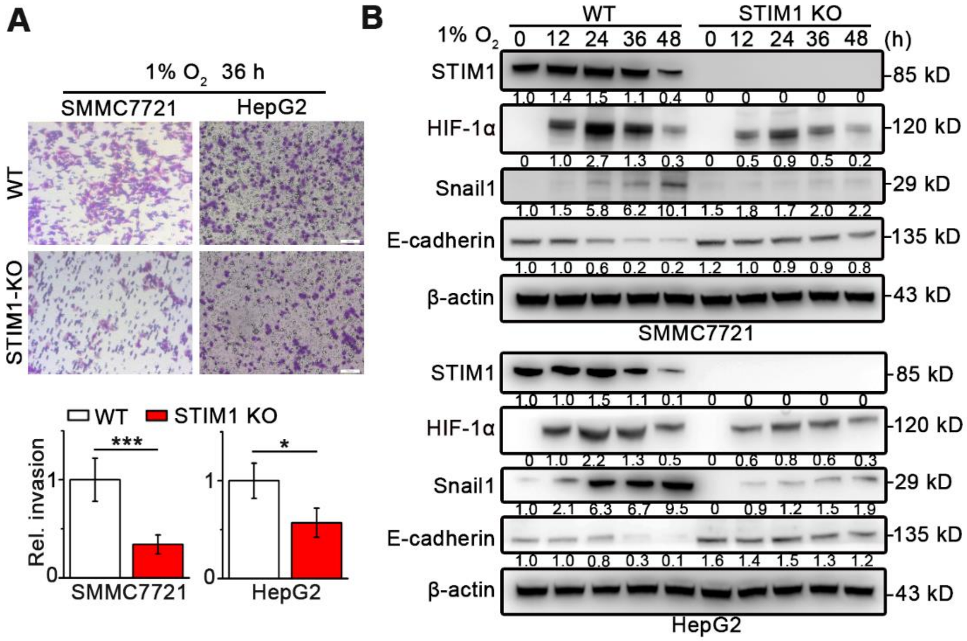

Figure S4. Deletion of STIM1 attenuates hypoxia-induced invasion and Snail1 expression. (**A**) Transwell assays were applied to evaluate invasion capabilities in WT- and STIM1 KO-SMMC7721 or HepG2 cells under hypoxia. (**B**) STIM1, HIF-1α, Snail1 and E-cadherin protein levels were measured in WT- and STIM1 KO-SMMC7721 or HepG2 cells cultured in hypoxia condition (1% O_2) with different time intervals. Data are expressed as mean ± SEM (n = 3). **p* < 0.05, ****p* < 0.001.

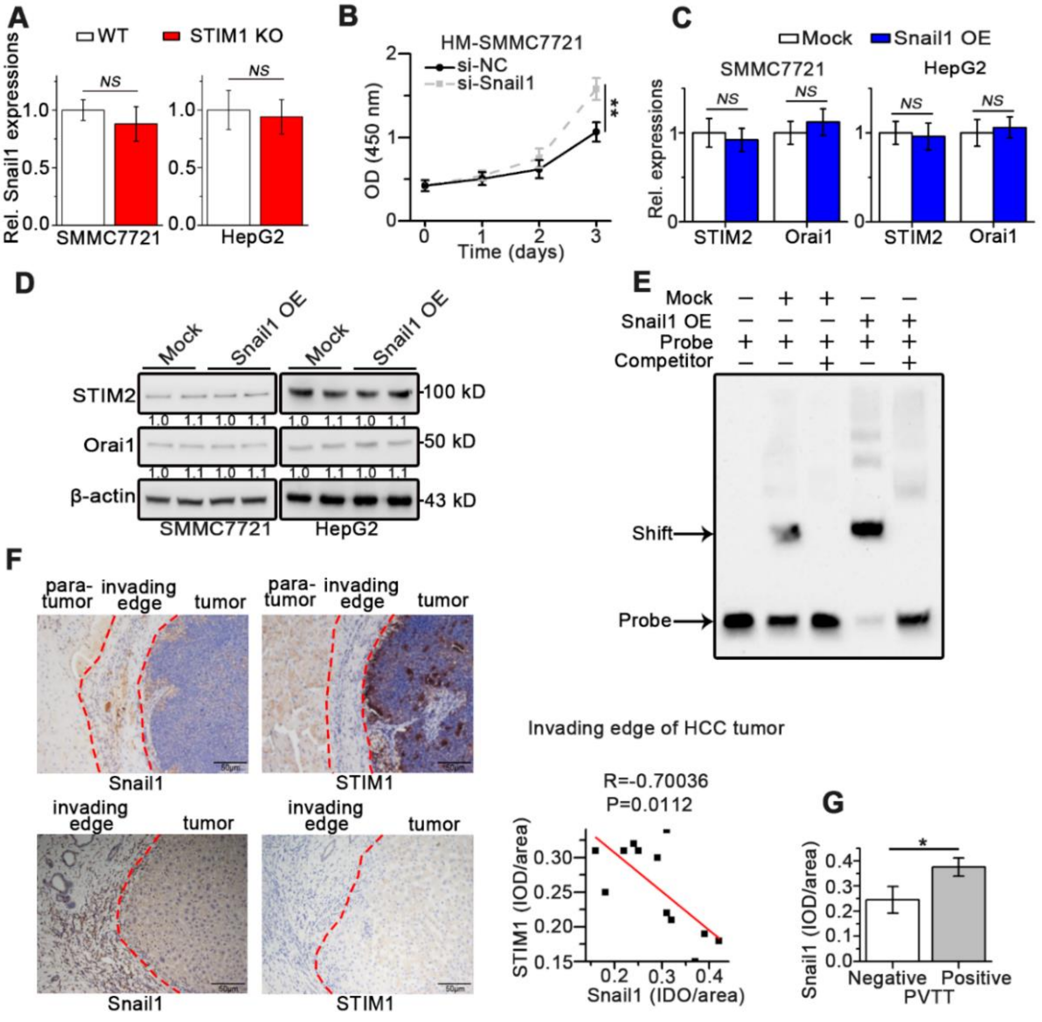

Figure S5. The interaction between STIM1 and Snail1 in HCC.

(**A**) Snail1 mRNA expressions in WT- and STIM1 KO-SMMC7721 or HepG2 cells. (**B**) The proliferation of HM-SMMC7721 subline transfected with si-NC or si-Snail1. (**C**, **D**) RT-qPCR (**C**) and WB (**D**) were used to assess STIM2 and Orai1 expressions in mock- and Snail1 OE-SMMC7721 and HepG2 cells. (**E**) EMSA assay was applied to detect the binding with Snail1 and *STIM1* promoter. Nuclear extracts prepared from mock- or STIM1 OE-SMMC7721 cells were incubated with the biotin-labeled probe corresponding to the E-box region in *STIM1* promoter, 100-fold excess of unlabeled probe was used as competitor, band shifts and free probes are indicated with black arrows. (**F**) Representative micrographs (400×) of immunohistochemical analysis STIM1 and Snail1 in tumor invading-edge from 12 HCC patients, and statistical analysis correlation between the IOD of Snail1 and STIM1 against IgG in the tumor invading-edge. (**G**) IOD of Snail1 against IgG in the tumor invading-edge of PVTT positive (n = 4) and PVTT negative (n = 8) HCC samples. Data are expressed as mean ± SEM (n = 3). *$p < 0.05$, **$p < 0.01$, NS represents no significant difference.

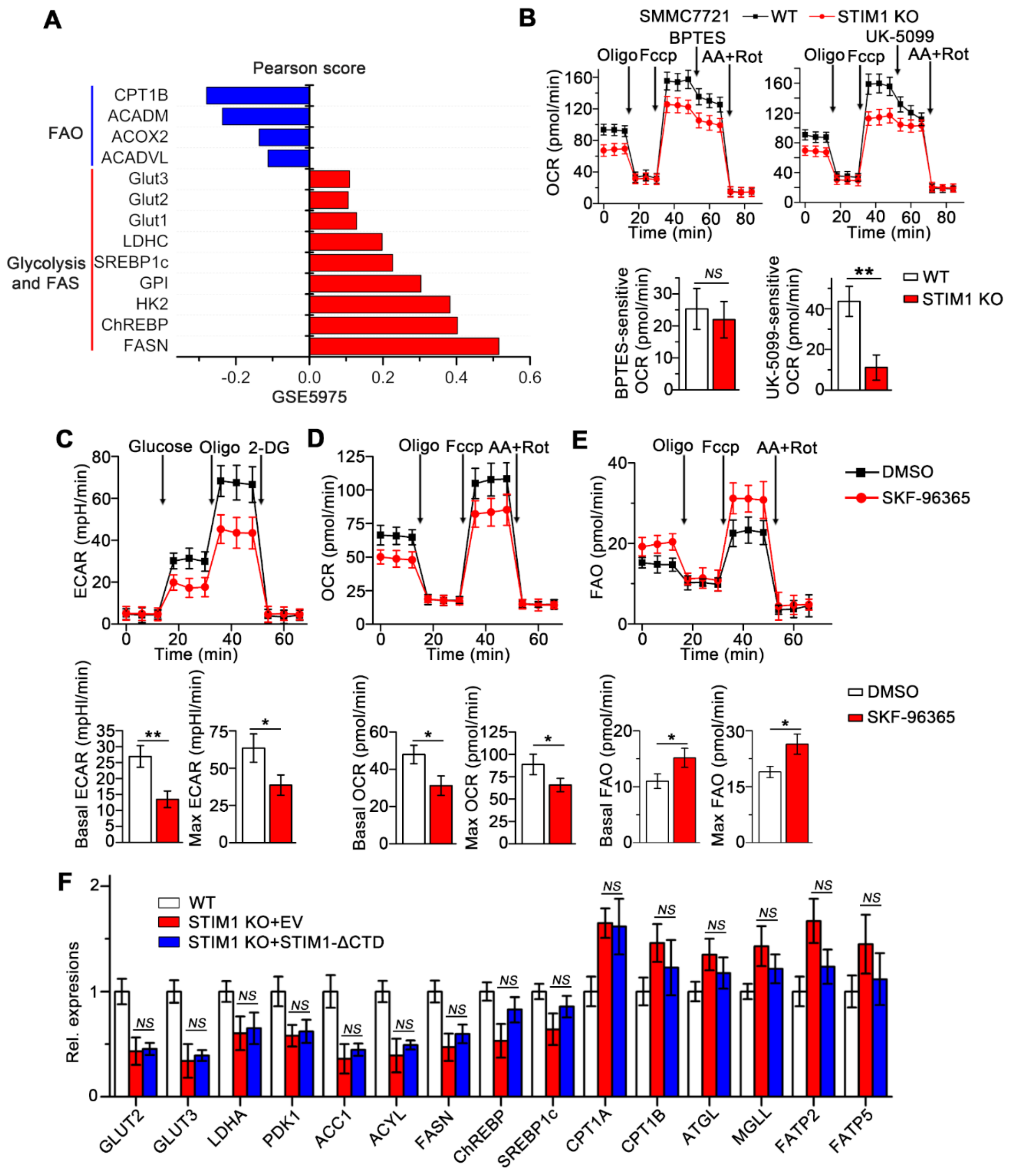

Figure S6. STIM1 programs metabolism in HCC cells *via* SOCE.

(**A**) Pearson correlation coefficient analysis was performed to determine the correlation between STIM1 and selected metabolic related genes (Pearson's correlation coefficient > 0.1 or < -0.1, *$p < 0.05$), microarray data was obtained from GSE5975. (**B**) Effects of BPTES (10 µM) or UK-5099 (150 µM) on OCR of WT- or STIM1 KO-SMMC7721 cells. (**C-E**) ECAR (**C**), OCR (**D**), FAO (**E**) caused by SFK-96365 (5 µM) treatment in SMMC7721 cells. (**F**) Indicated gene expressions in WT-, STIM1 KO+EV-, and STIM1 KO+STIM1-ΔCTD-SMMC7721 cells. Data are expressed as mean ± SEM (n = 3). *$p < 0.05$, **$p < 0.01$, NS represents no significant difference.

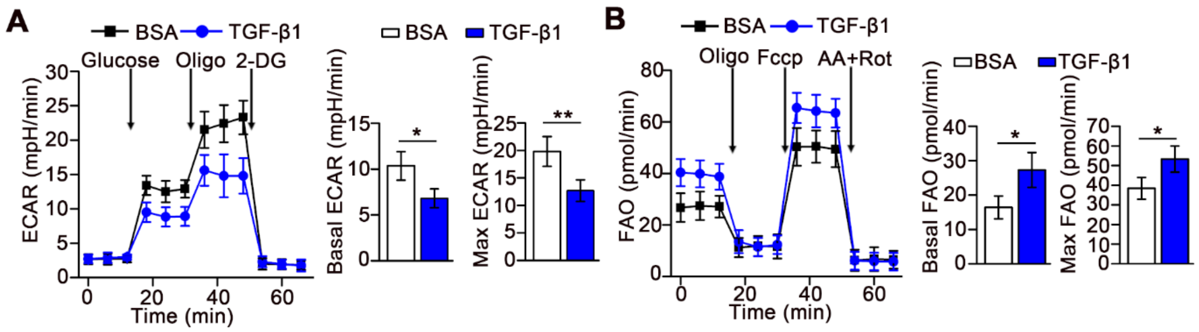

Figure S7. Metabolic switch triggered by TGF-β1 in SMMC7721 cells.

(**A**, **B**) ECAR (**A**) and FAO (**B**) of SMMC7721 cells after treated with TGF-β1 (20 ng/mL) for 48 h, and the BSA-treated group was used as control. Data are expressed as mean ± SEM (n = 3). *$p < 0.05$, **$p < 0.01$.

Supplementary Tables

Table S1. Clinicopathological characteristics of 12 HCC patients.

Patient No.	Age	Sex	Tumor Size(cm*cm*cm)	Procedure	Edmondson Grade	TNG Stage	Cirrhosis	HBsAg	PVTT
1	53	M	2.5*2*2.5	Surgical resection	Ⅰ	T1 N0 M0	No	Negative	Negative
2	47	F	3*3.5*4	Surgical resection	Ⅱ	T1 N0 M0	No	Negative	Positive
3	61	F	1.5*2*3	Surgical resection	Ⅰ	T1 N0 M0	Yes	Positive	Negative
4	54	M	3*3.5*5	Surgical resection	Ⅱ	T1 N0 M0	No	Positive	Positive
5	43	F	5*1.5*4	Surgical resection	Ⅱ	T1 N0 M0	Yes	Positive	Negative
6	62	M	3.5*2.5*5	Surgical resection	Ⅱ	T1 N0 M0	Yes	Negative	Positive
7	57	M	5*3*6.5	Surgical resection	Ⅲ	T1 N0 M0	No	Positive	Negative
8	47	F	3.5*4.3*5	Surgical resection	Ⅲ	T1 N0 M0	No	Positive	Negative
9	53	M	2.5*3*4	Surgical resection	Ⅰ	T1 N0 M0	No	Negative	Negative
10	58	M	5*7.5*9	Surgical resection	Ⅱ	T1 N0 M0	Yes	Positive	Negative
11	61	M	1.5*2*3.5	Surgical resection	Ⅱ	T1 N0 M0	No	Positive	Positive
12	57	F	2*3.5*5	Surgical resection	Ⅲ	T1 N0 M0	Yes	Negative	Negative

Table S2. Primers for RT-qPCR.

Gene	Species	Accession	Sequence of forward primer	Sequence of reverse primer
STIM1	Homo sapiens	NM_003156	TTGTCCATGCAGTCCCCTAG	GGTAGTGGTGATGGTGGTGA
STIM2	Homo sapiens	NM_001169117	AGACAACAATGTCAAAGGAACGA	ACTCCGGTCACTGATTTTCAAC
ORAI1	Homo sapiens	NM_032790	GGACGCTGACCACGACTAC	GGGACTCCTTGACCGAGTT
SNAI1	Homo sapiens	NM_005985	ACTGCAACAAGGAATACCTCAG	GCACTGGTACTTCTTGACATCTG
SLUG	Homo sapiens	NM_003068	CGAACTGGACACACATACAGTG	CTGAGGATCTCTGGTTGTGGT
CDH1	Homo sapiens	NM_004360	GCACCTTCCATGACAGACCC	GAGAACGCATTGCCACATACAC
GLUT1	Homo sapiens	NM_006516	CTCATCGCCCAGGTGTTC	TTCTCCTCGTTGCGGTTG
GLUT2	Homo sapiens	NM_000340	GGGCAATTATGATCTGTGGCA	TTCTGCTCACTCGATGCTTCT
GLUT3	Homo sapiens	NM_006931	GCTGGGCATCGTTGTTGGA	GCACTTTGTAGGATAGCAGGAAG
GLUT4	Homo sapiens	NM_001042	TGGGCGGCATGATTTCCTC	GCCAGGACATTGTTGACCAG
HK2	Homo sapiens	NM_000189	GGCTTGGAGCCACCACTCACC	CCTTCTGGAGCCCATTGTCCGT
HK3	Homo sapiens	NM_002115	GTGAGGTTGGGCTAGTTGTAGA	GTCCAGGGTATGGTCGAAGGT
LDHA	Homo sapiens	NM_001165415	TTGACCTACGTGGCTTGGAAG	GGTAACGGAATCGGGCTGAAT
LDHB	Homo sapiens	NM_002300	TCTGTGACCGCCAATTCTAAGA	GCACCAGATTGAGCCGACTC
LDHC	Homo sapiens	NM_002301	AGAACATGGTGATTCTAGTGTGC	ACAGTCCAATAGCCCAAGAGG
PDK1	Homo sapiens	NM_002610	GAGAGCCACTATGGAACACCA	GGAGGTCTCAACACGAGGT
PKM2	Homo sapiens	NM_182471	ATGTCGAAGCCCCATAGTGAA	TGGGTGGTGAATCAATGTCCA
PFKL	Homo sapiens	NM_002626	GCTGGGCGGCACTATCATT	TCAGGTGCGAGTAGGTCCG
PGAM1	Homo sapiens	NM_002629	TCTGGAGGCGCTCCTATGAT	TCTGTGAGGTCTGCATACCTG
PGK1	Homo sapiens	NM_000291	GACCTAATGTCCAAAGCTGAGAA	CAGCAGGTATGCCAGAAGCC
GPI	Homo sapiens	NM_001184722	CAAGGACCGCTTCAACCACTT	CCAGGATGGGTGTGTTTGACC
ENO1	Homo sapiens	NM_001201483	TGGTGTCTATCGAAGATCCCTT	CCTTGGCGATCCTCTTTGG
ALDOA	Homo sapiens	NM_000034	ATGCCCTACCAATATCCAGCA	GCTCCCAGTGGACTCATCTG
TPI	Homo sapiens	NM_000365	CTCATCGGCACTCTGAACG	GCGAAGTCGATATAGGCAGTAGG
FH	Homo sapiens	NM_000143	CCTGTGCATCCCAACGATCAT	AATTCCTGCCCAAGAGTAAGTG
CS	Homo sapiens	NM_004077	GGTGGCATGAGAGGCATGAA	TAGCCTTGGGTAGCAGTTTCT
SDH1	Homo sapiens	NM_003000	GGTGGCACAGTCAGCCTCGT	GGAGACCTAAAGCACCTGAAGACG
CD36	Homo sapiens	NM_000072	AAGCCAGGTATTGCAGTTCTTT	GCATTTGCTGATGTCTAGCACA
FABP1	Homo sapiens	NM_001443	AAGACAGTGGTTCAGTTGGAAG	TGAGTTCGGTCACAGACTTGAT
FATP2	Homo sapiens	NM_003645	TACTCTTGCCTTGCGGACTAA	CCGAAGCAGTTCACCGATATAC
FATP5	Homo sapiens	NM_012254	CATGGCGTGACAGTGATCCT	CAGCCCGTAGTCCATTGCC
LDLR	Homo sapiens	NM_000527	ACGGCGTCTCTTCCTATGACA	CCCTTGGTATCCGCAACAGA
VLDLR	Homo sapiens	NM_003383	CTGGGTATGCGACGATGATG	CTTGGTGTGTATGACTGGCTG
ACC1	Homo sapiens	NM_000664	ACCACCAATGCCAAAGTAGC	CTGCAGGTTCTCAATGCAAA
ACYL	Homo sapiens	NM_001096	CAGCATCGCAAACTTCAC	TGGTCTTCCCGACTTCTC
FASN	Homo sapiens	NM_004104	CAAAGAAGCCCATCTCCCG	GCTGCTCCACGAACTCAAACA
SCD	Homo sapiens	NM_005063	GCCCCTCTACTTGGAAGACGA	AAGTGATCCCATACAGGGCTC
CPT1A	Homo sapiens	NM_001031847	ATCAATCGGACTCTGGAAACGG	TCAGGGAGTAGCGCATGGT
CPT1B	Homo sapiens	NM_152246	ATCATGGCGTGGATGATGT	CCTCTCATGGTGAACAGCAA
ACADVL	Homo sapiens	NM_000018	ACGGGCGTACTGGGTGTT	ATGGTGGAGGAGACCACTTG
LACD	Homo sapiens	NM_001608	TGCAATAGCAATGACAGAGCC	CGCAACTACAATCACAACATCAC
MCAD	Homo sapiens	NM_000016	CGCAACTACAATCACAACATCAC	AGCTCCGTCACCAATTAAAACAT
HSL	Homo sapiens	NM_005357	GAAGGCTATGTTGTCCTCCG	ATGAGAAAACCAGTGCTCGG
ATGL	Homo sapiens	NM_020376	ACCTCAATGAACTTGGCACC	CAACGCCACGCACATCTA
MGLL	Homo sapiens	NM_001003794	AATGCAGACGGACAGTACCTC	GAGCCAGCTCTTCATAGCGG
UCP2	Homo sapiens	NM_003355	TACGTCCCAGGAGATGGAGA	CCGTGAGACCTTACAAAGCC
ACOX1	Homo sapiens	NM_007292	GGAACTCACCTTCGAGGCTTG	TTCCCCTTAGTGATGAGCTGG
NDUFA	Homo sapiens	NM_004541	GCGTACATCCACAGGTTCACT	GCGCCTATCTCTTTCCATCAGA
UQCRC1	Homo sapiens	NM_003365	GGGGCACAAGTGCTATTGC	GTTGTCCAGCAGGCTAACC
HMGCR	Homo sapiens	NM_000859	TTCTTGCCAACTACTTCGTGTT	GCTGCCAAATTGGACGACC
SREBP1c	Homo sapiens	NM_004176	ACAGTGACTTCCCTGGCCTAT	GCATGGACGGGTACATCTTCAA
ChREBP	Homo sapiens	NM_032951	CGACCCCACACTCACACGCC	GCCACACACGGGGCTCTTCC
PPARA	Homo sapiens	NM_005036	AGAGTGGGCTTTCCGTGTC	GCCGCCTTCAGGTACAGTAG
PPARG	Homo sapiens	NM_005037	GGGATCAGCTCCGTGGATCT	TGCACTTTGGTACTCTTGAAGTT
PPARGC1A	Homo sapiens	NM_013261	CTGCGGGATGGAGACAG	TTCGTTTGACCTGCGCAAAG
ACTB	Homo sapiens	NM_001101	GCGCGGCTACAGCTTCA	CTTAATGTCACGCACGATTTCC

Table S3. Antibody used for WB, ChIP and IHC.

Antibodies	Source	Identifier
STIM1 (for WB and IHC)	Cell Signaling Technology	Ca# 5668, RRID:AB_10828699
E-cadherin	Cell Signaling Technology	Cat# 3195, RRID:AB_2291471
Snail1 (for WB, ChIP and EMSA)	Cell Signaling Technology	Cat# 3879, RRID:AB_2255011
HIF-1α	Cell Signaling Technology	Cat# 36169, RRID:AB_2799095
p-CaMKII (Thr286)	Cell Signaling Technology	Cat# 12716, RRID:AB_271388
HK2	Cell Signaling Technology	Cat# 2106, RRID:AB_823520
LDHA	Cell Signaling Technology	Cat# 3582, RRID:AB_2066887
CPT1α	Cell Signaling Technology	Ca# 12252
AMPK and ACC Antibody Sampler Kit	Cell Signaling Technology	Cat# 9957, RRID:AB_823677
p-LKB1	Cell Signaling Technology	Cat# 3482, RRID:AB_2198321
LKB1	Cell Signaling Technology	Cat# 3047, RRID:AB_2198327
GSK-3 Antibody Sampler Kit	Cell Signaling Technology	Cat# 9369, RRID:AB_1196648
PKM2	Cell Signaling Technology	Cat# 4053, RRID:AB_1904096
PFKL	Cell Signaling Technology	Cat# 8164, RRID:AB_2713957
PPARγ	Cell Signaling Technology	Cat# 2443, RRID:AB_823598
AKT	Cell Signaling Technology	Cat# 9272, RRID:AB_329827
p-AKT	Cell Signaling Technology	Cat# 9271, RRID:AB_329825
FASN	Cell Signaling Technology	Ca# 3180
Ubiquitin	Cell Signaling Technology	Ca# 3936
Rabbit IgG (for ChIP and IHC)	Cell Signaling Technology	Ca# 3900
Glut3	Abcam	Cat# ab41525, RRID:AB_732609
STIM2	Abcam	Cat# ab59342, RRID:AB_945663
Orai1	Abcam	Ca#: ab244352
ChREBP	Abcam	Cat# ab92809, RRID:AB_10562135
PPAR-α	Abcam	Cat# ab24509, RRID:AB_448110
SREBP1c	Abcam	Cat# ab28481, RRID:AB_778069
Snail1 (for IHC)	Santa Cruz Biotechnology	Cat# 393172
β-actin	Beyotime	Cat# AF1681
